# A Terphenyl Supported Dioxophosphorane Dimer: the Light Congener of Lawesson's and Woollins’ Reagents

**DOI:** 10.1002/chem.202200376

**Published:** 2022-04-06

**Authors:** Laura E. English, Aleksandra Pajak, Claire L. McMullin, John P. Lowe, Mary F. Mahon, David J. Liptrot

**Affiliations:** ^1^ Department of Chemistry University of Bath Bath BA2 7AY UK; ^2^ Centre for Sustainable and Circular Technologies Bath BA2 7AY UK

**Keywords:** arene ligands, chalcogens, organocatalysis, phosphorus heterocycles

## Abstract

Thermolysis of a 1,3‐dioxa‐2‐phospholane supported by the terphenyl ligand Ar^iPr4^ (Ar^iPr4^=[C_6_H_3_‐2,6‐(C_6_H_3_‐2,6‐iPr_2_)]) at 150 °C gives [Ar^iPr4^PO_2_]_2_ via loss of ethene. [Ar^iPr4^PO_2_]_2_ was characterised by X‐ray crystallography and NMR spectroscopy; it contains a 4‐membered P−O−P−O ring and is the isostructural oxygen analogue of Lawesson's and Woollins’ reagents. The dimeric structure of [Ar^iPr4^PO_2_]_2_ was found to persist in solution through VT NMR spectroscopy and DOSY, supported by DFT calculations. The addition of DMAP to the 1,3‐dioxa‐2‐phospholane facilitates the loss of ethene to give Ar^iPr4^(DMAP)PO_2_ after days at room temperature, with this product also characterised by X‐ray crystallography and NMR spectroscopy. Replacement of the DMAP with pyridine induces ethene loss from the 1,3‐dioxa‐2‐phospholane to provide gram‐scale samples of [Ar^iPr4^PO_2_]_2_ in 75 % yield in 2 days at only 100 °C.

## Graphical abstract

Pyridines have been shown to mediate the release of ethene from a terphenyl supported 1,3‐dioxo‐2‐phospholane. This generates a dioxophosphorane dimer which is an oxygen analogue of Lawesson's and Woollins’ reagents. This compound was structurally characterised and its solution behaviour interrogated. Insight into the ethene release reaction is provided by the isolation of a DMAP‐coordinated dioxophosphorane

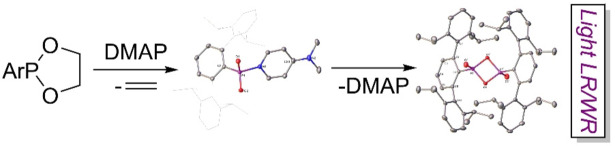

Chalcogens of phosphorus are widely utilised reactive molecules, finding applications in diverse fields such as desiccation,^[1]^ chalcogenation^[2]^ and as ligands.^[3]^ Their utility and ubiquity has even resulted in the development of eponymous compounds such as Lawesson's (LR)^[4]^ and Woollins’ reagents (WR)^[5]^ ArP(=X)(μ_2_‐X)_2_P(=X)Ar (LR: Ar=4‐MeOC_6_H_4_, X=S; WR: Ar=Ph, X=Se); aryl phosphorus disulfide and diselenide respectively. These reagents have been intensively studied, and widely applied in the thiolation^[6]^ and selenation^[7]^ of carbonyl compounds, amongst other related reactions. Mechanistically, these reactions have been proposed to proceed via the formation of ArPX_2_ monomers which react with C=O bonds to form C=X and [ArP(O)X]_n_ products, the latter containing strong P−O bonds which provide a reaction driving force. One such ArPX_2_ monomer (Ar=2,4,6‐^t^Bu_3_C_6_H_2_, X=S) was reported by Appel and co‐workers in 1983.^[8]^

In contrast to the heavier congeners, no ArP(=O)(μ_2_‐O)_2_P(=O)Ar system has been structurally characterised. Instead, [ArPO_2_]_3_ trimers, as well as higher oligomers and polymers have been observed.^[9]^ The monomeric [RPO_2_] fragment itself is highly reactive, likely a consequence of its unsaturated σ^3^λ^5^ structural arrangement and the limited steric demand provided by the lightest chalcogen, oxygen. In light of this potential, there have been forays into the generation of [RPO_2_] species. The heavy pnictogen analogues of nitromethane, MePO_2_,^[10]^ and nitrobenzene, PhPO_2_,^[11]^ have been detected via matrix isolation infrared spectroscopy. This method has also been used to characterise the monomeric forms of LR^[12]^ and WR^[13]^ via infrared and UV/Vis spectroscopy. Coordinative saturation by Lewis base adduction has been used to isolate related species. In this vein the oxidation of ArP(NHC) to Ar(NHC)PO_2_ has begun to emerge as a generalisable route towards coordinated RPO_2_ fragments which are isolable under an inert atmosphere.^[14]^

Alongside these oxidative methods, a number of other routes have been described to generate ArPO_2_ in situ. Flash vacuum pyrolysis of aryl substituted 1,3‐dioxa‐2‐phospholanes, ArP(OCH_2_)_2_ (Ar=Ph, 2‐Ph−Ph, 2,4,6‐^t^Bu_3_C_6_H_2_), has been reported to generate aryldioxophosphoranes via loss of ethene, although the harsh conditions involved often result in decomposition of the −PO_2_ moiety.^[15]^ Cyclic phosphonates have been employed as starting materials in the generation of base‐stabilised aryldioxophosphoranes by the way of the direct reaction between DMAP (DMAP=NC_5_H_4_‐4‐N(CH_3_)_2_) and [PhPO_2_]_3_ to yield Ph(DMAP)PO_2_. This compound reacted with B(C_6_F_5_)_3_ to yield the “push‐pull” adduct Ph(DMAP)P(=O)O−B(C_6_F_5_)_3_.^[9]^

In the flash vacuum pyrolysis of 1,3‐dioxa‐2‐(2,4,5‐tri‐*tert*‐butylphenyl)‐2‐phospholane, the observed product was thought to be the result of activation of a flanking *tert*‐butyl group by the highly reactive dioxophosphorane.^[15b]^ This reactivity is often observed for aryl ligands substituted with large alkyl groups as a consequence of the localisation of these groups in the coordination sphere of the reactive centre, which is also the origin of their steric protection (Scheme 1).

**Scheme 1:**
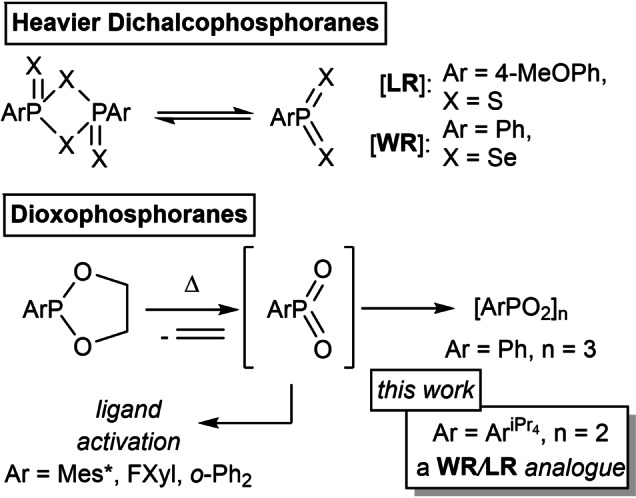
The synthesis and reactivity of a range of dichalcophosphoranes. Mes*=2,4,6‐(tBu)_3_‐C_6_H_2_; FXyl=2,6‐(F_3_C)_2_‐C_6_H_3_; *o*‐Ph_2_=2‐Ph‐C_6_H_4_.

Over the last three decades, terphenyls have emerged as pre‐eminent ligands in the stabilisation of reactive main group centres as a consequence of their flanking steric bulk which serves to prevent access to the ligated main group atom without orienting groups directly within its coordination sphere. More recently, there has been an increasing awareness of the extent to which attractive dispersion forces between the ligands in terphenyl derivatives contributes to their structures and stability.^[16]^ Recent work has shown that the action of DMAP upon a 1,3‐dioxa‐2‐elementolane derivative, LMgB(OCMe_2_)_2_, can induce the ejection of alkenes and generate LMgBO_2_(DMAP) (L=[HC{H_3_CCN(2,6‐iPr_2_‐C_6_H_3_)}_2_]).^[17]^ We thus set out to investigate the capacity of terphenyl ligands to support dioxophosphoranes and of Lewis bases to induce ethene loss from substituted 1,3‐dioxa‐2‐phospholanes in the hope that access via mild conditions would prevent unwanted side reactivity of the ‐PO_2_ moiety. We herein report the reaction of Ar^iPr4^P(OCH_2_)_2_ with either pyridine or DMAP affords the evolution of ethene at a reduced temperature relative to thermolysis and provides access to a light analogue of LR and WR, Ar^iPr4^P(=O)(μ_2_‐O)_2_P(=O)Ar^iPr4^.

We began by synthesising Ar^iPr4^P(OCH_2_)_2_, **1**, via the reaction of ClP(OCH_2_)_2_ and Ar^iPr4^Li, the identity of which was confirmed by multinuclear NMR spectroscopy, mass spectrometry and single crystal X‐ray crystallography (See Supporting Information). The thermolysis of **1** was then investigated by differential scanning calorimetry. This analysis indicated an event consistent with melting at ca. 150 °C followed by a steady exothermic reaction which continues to ca. 270 °C containing a sharp endothermic kink. Notably, a second calorimetric run on this sample was bereft of any such features. We interpreted this as implication that an irreversible reaction had occurred, and rationalised the endothermic kink as a measurement artefact reflecting the loss of a gas (See Supporting Information, Figure S2).

We thus thermolyzed a neat sample of **1** in an NMR tube at 150 °C hoping to identify the product of this reaction. After 36 h, the reaction mixture was dissolved in C_6_D_6_ and analysed by ^1^H and ^31^P NMR spectroscopy. The ^1^H NMR spectrum suggested the presence of one major new Ar^iPr4^ containing system as well as ethene, identified by a peak at 5.25 ppm (see Figure 1),^[18]^ which may have been trapped in the solid during thermolysis and liberated upon dissolution.

**Figure 1:**
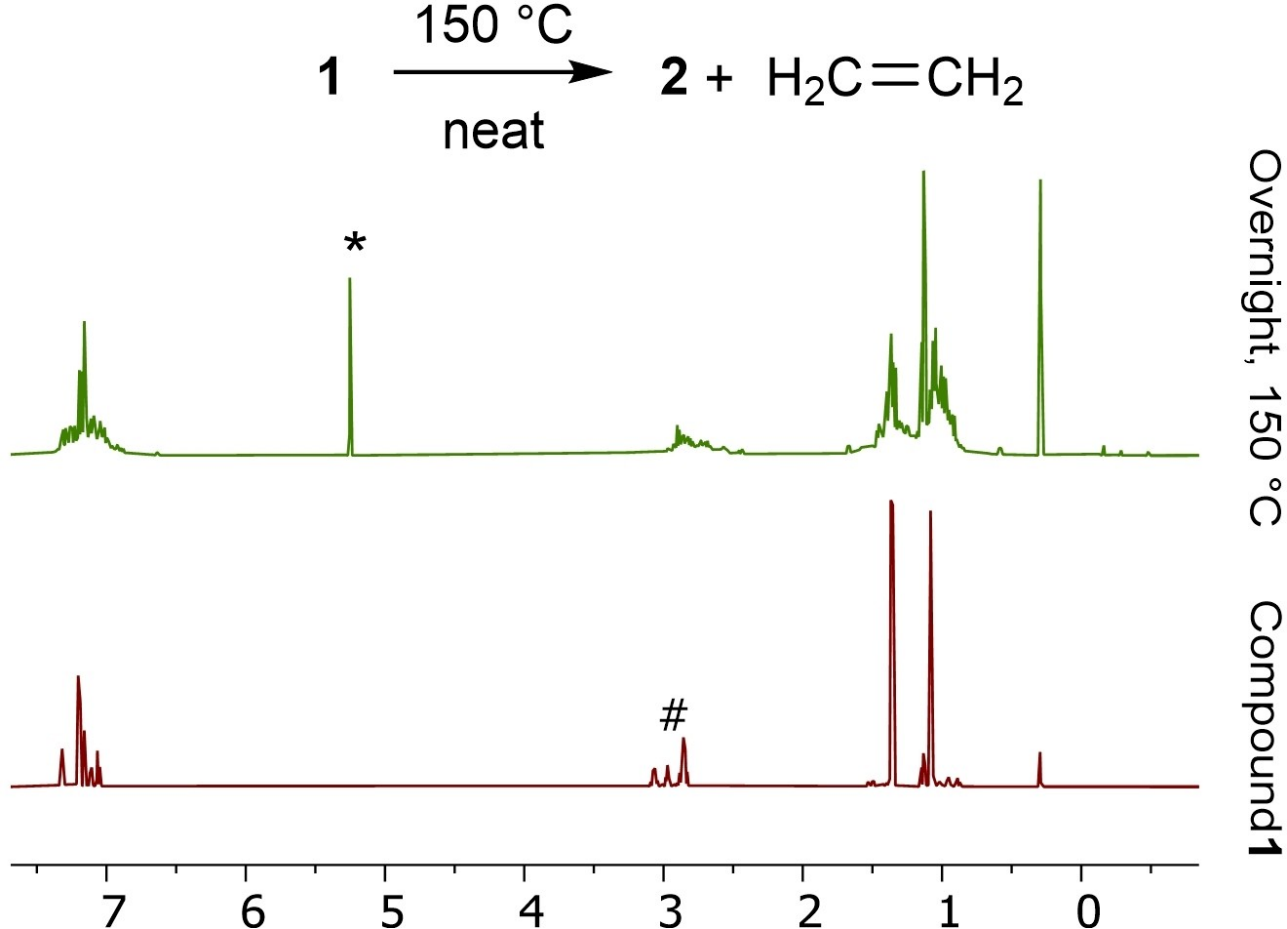
A scheme and stacked partial ^1^H NMR spectra representing the thermolysis of compound **1**, thermolysis performed neat at 150 °C, NMR spectroscopy performed in C_6_D_6_, * H_2_C=CH_2_, # −CH_2_CH_2_− in **1**

Furthermore, the resonances associated with the (−CH_2_CH_2_−) bridge of **1** were absent. The ^31^P NMR spectrum contained a major resonance at 17.5 ppm (See Supporting Information, Figures S3 and S4). Recrystallisation of the reaction mixture from C_6_D_6_ provided material suitable for single crystal X‐ray crystallography (Figure 2) confirming the loss of ethene from **1** and the generation of a new phosphorus oxide, **2**.

**Figure 2:**
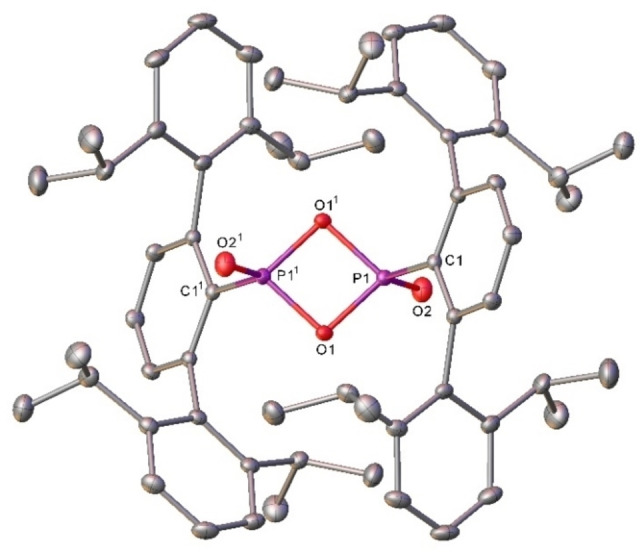
Molecular structure (30 % probability ellipsoids) of compound **2**. Hydrogen atoms are omitted for clarity. Selected bond lengths (Å) and angles (°) C1‐P1 1.8011(10); P1‐O1 1.6391(8); P1‐O2 1.4442(9); P1‐P1^1^ 2.3905(5); C1‐P1‐O1 108.30(4); C1‐P1‐O2 116.24(5); P1‐O1‐P1^1^ 93.65(4); O1‐P1‐O1^1^ 86.35(4). Atoms with superscripted labels are generated by the 1/2 ‐*x*, ^3^/_2_‐*y*, 1‐*z*

Compound **2** is a dimeric, terphenyl‐supported dioxophosphorane and as the first such 4‐membered P^V^ ring structurally characterised, it constitutes the light analogue of LR and WR. **2** can be considered a dimer of Ar^iPr4^PO_2_ (Figure 3) containing two P−O−P bridges comprising single bonds (P‐μ_2_‐O=1.6391(8) Å) as well as a terminal P=O fragment on each phosphorus atom (P=O_terminal_=1.4442(9) Å) which are *trans*‐disposed across the P−O−P−O ring. The phosphorus chalcogen bonds are, unsurprisingly, shorter than those found in an unsubstituted analogue of LR^[19]^ and in WR,^[20]^ reflecting the larger size and weaker bonding found for the heavier chalcogens, whilst the P−X−P angles are similar in all three cases (X=O, 86.35(4) in **2**; S, 86.58(5);^[19b]^ Se, 85.45(9)°^[20]^). In comparison to [MesPO_2_]_3_, the P−O bond lengths were similar however, the P−O−P angles were significantly narrower, ([MesPO_2_]_3_ P−O−P=101.2° on average).^[9]^

**Figure 3:**
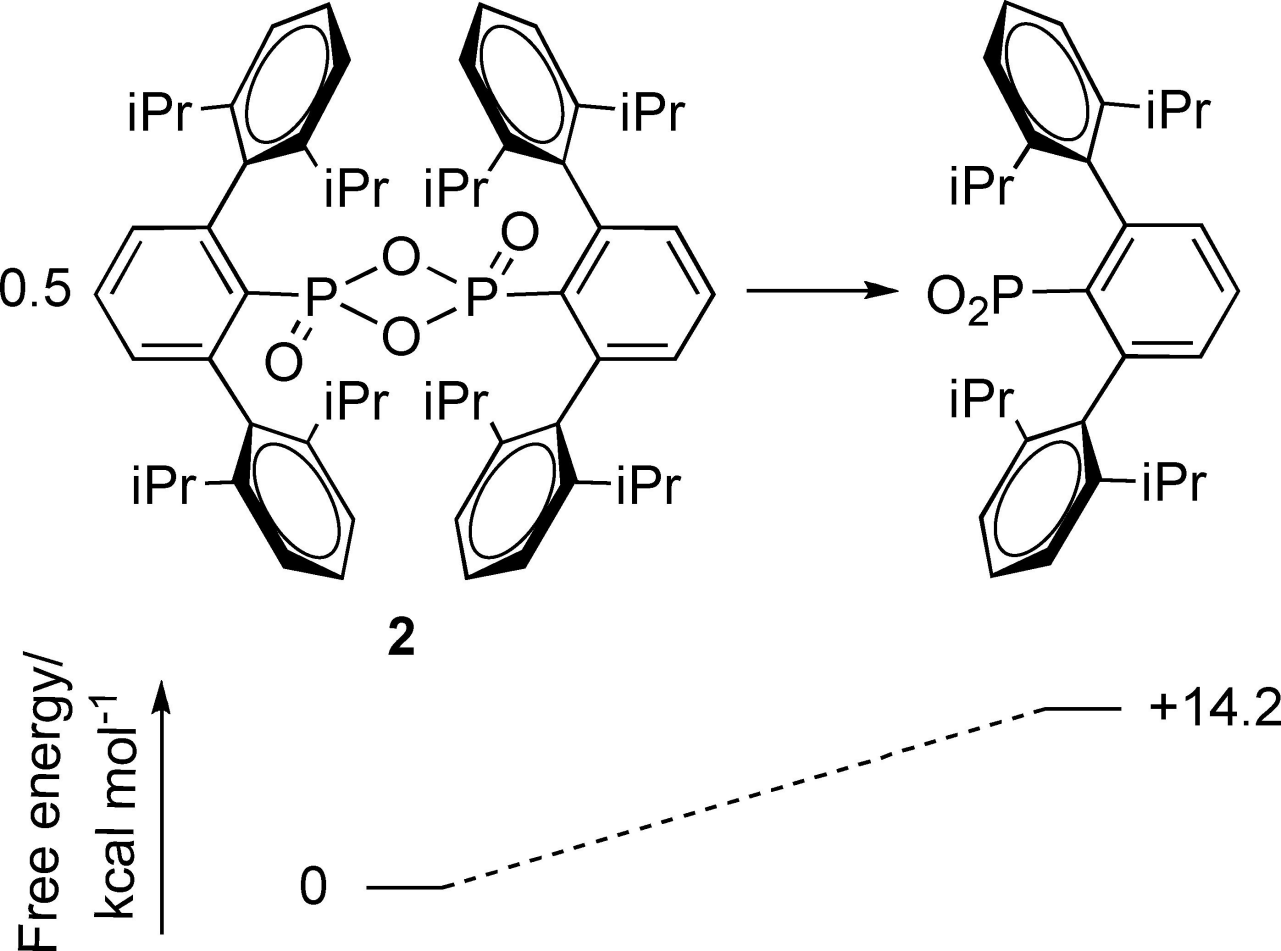
A skeletal structure of compound **2**, and its corresponding monomer with DFT calculated free energies (BP86‐D3BJ(C_6_H_6_)/BS2//BP86/BS1) for its monomerization,

We then undertook studies to confirm the solution speciation of **2**. Its ^31^P NMR spectrum, taken in *d*_8_‐toluene, was unchanged with varied temperature between 298 and 358 K (See Supporting Information, Section 5). The ^1^H NMR DOSY of **2** provided a hydrodynamic radius of 6.14 Å which more closely correlated with the calculated radius of [Ar^iPr4^PO_2_]_2_ ([Ar^iPr4^PO_2_]_n_: n=1, r_calc_=4.70 Å; n=2, r_calc_=5.90 Å). These data were interpreted to indicate that **2** persisted as a dimer in solution (See Supporting Information, Sections 6 and 7).

Attempts were then made to trap any Ar^iPr4^PO_2_ present in solution via the addition of Lewis acidic and basic reagents, as has been shown for Woollins’ reagent.^[21]^ DFT calculations (BP86‐D3BJ(C_6_H_6_)/BS2//BP86/BS1, see Figure 3 and Supporting Information for full computational details) showed that the monomer was 14.2 kcal mol^−1^ higher in free energy than the dimer suggesting a very low concentration of this species, if any, was likely. Addition of an equivalent of B(C_6_F_5_)_3_ to a C_6_D_6_ solution of **2** provided no evidence of reaction, even with extended time and elevated temperature. In contrast, addition of an equimolar amount of DMAP to **2** provided two resonances in the ^31^P NMR spectrum; that of pure **2** and a resonance at 6.8 ppm (Figure 4a), which we attributed to the DMAP adduct of Ar^iPr4^PO_2_, Ar^iPr4^(DMAP)PO_2_ (Compound **3**). We interpret these data to suggest that rather than trapping free Ar^iPr4^PO_2_ that might be present in solution, the DMAP is capable of cracking the dimer in compound **2**, as we would expect any free Ar^iPr4^PO_2_ to react readily with both B(C_6_F_5_)_3_ and DMAP. Furthermore, no reaction was observed between **2** and a diene,^[22]^ which also indicates that the spontaneous monomerization of **2** is unlikely and that, essentially, no free Ar^iPr4^PO_2_ exists in solutions thereof.

**Figure 4:**
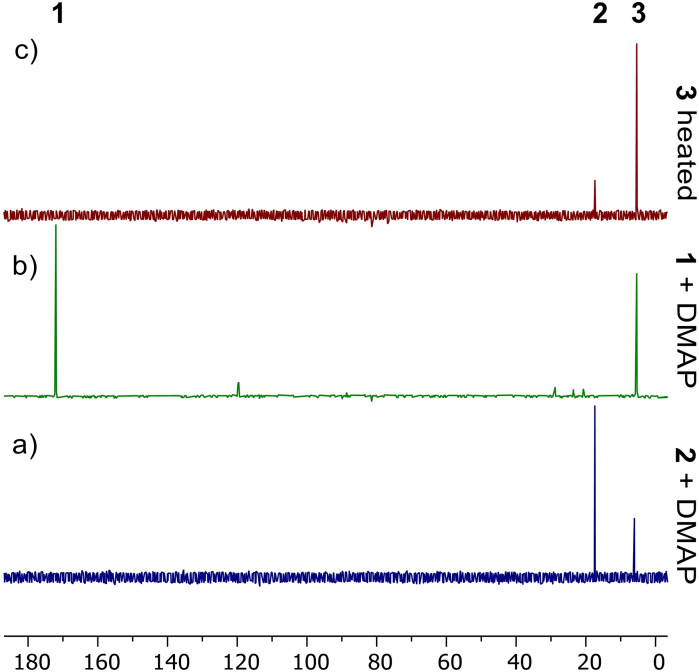
Representative ^31^P NMR spectra for a) the partial reaction of compound **2** with DMAP to generate compound **3**, b) the reaction of compound **1** with DMAP to form compound **3**, c) the effect of heating compound **3** to partially reform compound **2** and DMAP.

The NMR spectra of the reaction of compound **2** and DMAP contained evidence of compound **2**, free DMAP and compound **3** suggesting the former two compounds are in equilibrium with the latter. Nevertheless, repeated attempts to force the equilibrium completely towards compound **3** via heating were unsuccessful, yielding only mixtures of **2** and **3** according to the ^31^P NMR spectrum (See Supporting Information, Figures S5 and S6). In order to isolate an analytically pure sample of **3**, we investigated the in situ generation of **2** in the presence of DMAP. Addition of DMAP to **1** showed signs of ethene release even at room temperature on an NMR scale (See Figure 4b and Supporting Information, Figures S7 and S8).

On a gram‐scale, an equimolar mixture of **1** and DMAP left at room temperature in THF for 5 days resulted in the precipitation of a fine white material. This material was found to be analytically pure **3**, which was produced in 72 % yield and could be recrystallised from iso‐propanol to yield material suitable for SC‐XRD (Figure 5). The metric data for **3** are similar those to described for Ph(DMAP)PO_2_ by Manners and co‐workers.^[9]^ The P−O bond lengths are similarly short (P−O: **3**, 1.4738(8) and 1.4781(8); Ph(DMAP)PO_2_, 1.4752(17) and 1.4742(14) Å) indicating a degree of multiple bonding and the geometrical parameters^[23]^ for the P atoms align closely (τ_4_: **3**, 0.90; Ph(DMAP)PO_2_, 0.88) reflecting the similar, distorted tetrahedral configurations. Activation of the WR dimer by pyridine has also been reported to yield Ph(C_5_H_5_N)PSe_2_ ^[21]^ which shows much longer P−Se bonds (2.108(3), 2.106(3) Å) and marginally narrower Se−P−Se angles (120.30(9)°).

**Figure 5:**
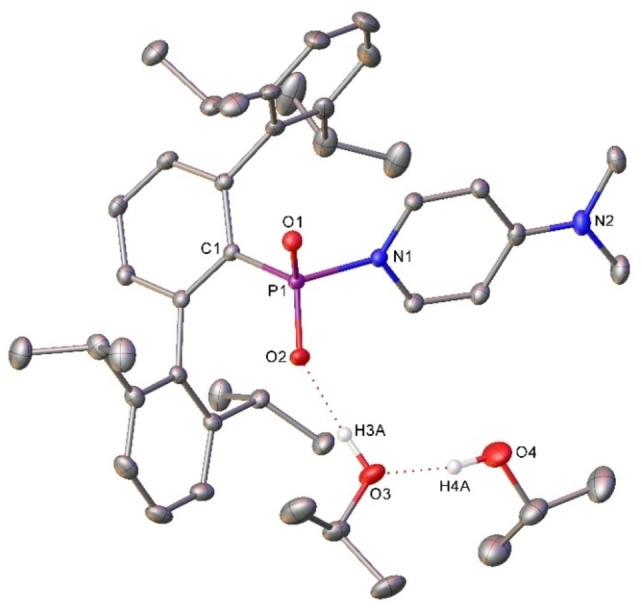
Molecular structure (30 % probability ellipsoids) of compound **3**. Hydrogen atoms except those attached O3 and O4 which are involved in hydrogen bonding are omitted for clarity. Selected bond lengths (Å) and angles (°) C1‐P1 1.8339(11); P1‐O1 1.4738(8); P1‐O2 1.4781(8); P1‐N1 1.8085(10); C1‐P1‐O1 110.35(5); C1‐P1‐O2 109.38(5); O1‐P1‐O2 122.99(5); C1‐P1‐N1 106.95(5); O1‐P1‐N1 103.51(5); O2‐P1‐N1 102.06(5).

Infrared spectroscopy was applied to compounds **2** and **3** (see Supporting Information, Figures S29 and S30). The ATR FTIR spectrum of **2** contains an absorption at 1182 cm^−1^ which was tentatively attributed to a P=O stretch, as well as a stretch at 799 cm^−1^ which was assigned as associated with the P−O−P−O ring based on its absence in the spectrum of **3**. This can be compared to similar modes reported for [PhP(O)O]_3_ by Manners and co‐workers, which occurred at 974 cm^−1^.^[9]^ The ATR FTIR spectrum of compound **3** contained a number of stretches present for **2** implying some dissociation of the DMAP in the solid state. It also contained a new absorbance at 1025 cm^−1^ which, if reflecting a P=O stretching mode, would imply marginally weaker P=O bonding in **3** compared to **2**, corresponding to the crystallographically defined P=O bond parameters.

Heating of **3** showed some evidence of DMAP decomplexation by NMR spectroscopy, through the reappearance of resonances associated with both **2** and free DMAP (See Figure 4c and Supporting Information, Figures S9 and S10). These results hinted that the release of DMAP from **3** was a facile reaction, and that **1** released ethene at lower temperatures in the presence of DMAP, suggesting that access to **2** via the extrusion of ethene from **1** catalysed by DMAP might be viable. Addition of 20 mol % DMAP to a solution of **1** in C_6_D_6_ followed by heating overnight at 100 °C provided firm evidence of this catalysis with complete loss of ethene observed, however **2** produced by this method was contaminated with **3** (See Supporting Information, Figures S11 and S12). We hoped that the replacement of DMAP by a less nucleophilic analogue, pyridine, might allow this transformation to occur in a more efficacious time frame but provide material which could be readily converted to **2**. Hence, we were thus extremely gratified to find that reaction of **1** with an equivalent of pyridine in C_6_D_6_ provided a good yield of analytically pure **2** in 4 days at 100 °C (See Supporting Information, Figures S13 and S14). This reaction was scaled up to 1 g in toluene, providing an isolated yield of 75 %, with a reduction of reaction time to 2 days.

Whilst **2** showed no propensity to react with boranes, we expected the terminal P−O moieties in **3** to readily undertake this reaction. Addition of one equivalent of B(C_6_F_5_)_3_ to a suspension of **2** in C_6_D_6_ gave rise to a number of resonances in the ^1^H, ^19^F and ^31^P NMR spectra (See Supporting Information, Figures S15‐S17). Full attribution of these peaks was not possible. These results are a contrast to Ph(DMAP)PO_2_ which cleanly formed an adduct, Ph(DMAP)P(=O)O−B(C_6_F_5_)_3_, with B(C_6_F_5_)_3_ and most likely reflect the extreme steric crowding in **3**. As noted, **3** is in equilibrium with **2** and DMAP, and it is likely that the borane reacts not only with the P−O bonds but as a sequestering agent for the DMAP driving the equilibrium towards **2** and resulting in mixtures of compounds.

In conclusion, we have developed a new method for the low‐temperature synthesis of [RPO_2_] moieties (Scheme 2). This approach has allowed us to extend the range of arenes supporting [ArPO_2_]_n_ systems to include terphenyls. Moreover, the exquisite control of the coordination environment provided by the Ar^iPr4^ ligand has facilitated the generation of a novel dimeric structure. The nucleophile‐catalysed release of ethene from 1,3‐dioxa‐2‐phospholanes provides convenient access to a light chalcogen congener of Lawesson's and Woollins’ reagents, Ar^iPr4^P(=O)(μ_2_‐O)_2_P(=O)Ar^iPr4^ (**2**), as well as its DMAP adduct, Ar^iPr4^(DMAP)PO_2_ (**3**). NMR spectroscopy supported by density functional theory calculations has provided evidence of the persistence of the dimeric nature of **2** in solution. We believe this new approach to generate dioxophosphoranes will enhance the range of such species accessible under mild conditions and lead, eventually, to the synthesis of an isolable monomeric RPO_2_ species as was established for a dithioxophosphorane almost forty years ago.^[8]^

**Scheme 2:**
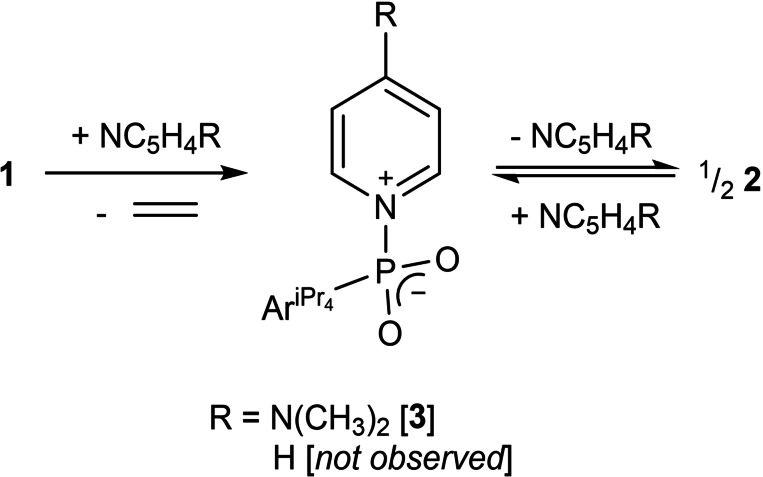
The organocatalysed release of ethene from a 1,3‐dioxa‐2‐phospholane to generate a dioxophosphorane dimer

Deposition Numbers 2149561 (for **1**), 2149562 (for **2**), 2149563 (for **3**) contain(s) the supplementary crystallographic data for this paper. These data are provided free of charge by the joint Cambridge Crystallographic Data Centre and Fachinformationszentrum Karlsruhe Access Structures service.

## Conflict of interest

The authors declare no conflict of interest.

## Supporting information

As a service to our authors and readers, this journal provides supporting information supplied by the authors. Such materials are peer reviewed and may be re‐organized for online delivery, but are not copy‐edited or typeset. Technical support issues arising from supporting information (other than missing files) should be addressed to the authors.

Supporting Information

## Data Availability

The data that support the findings of this study are available in the supplementary material of this article.
