# Supplementary material for: A Terphenyl Supported Dioxophosphorane Dimer: the Light Congener of Lawesson's and Woollins’ Reagents
Source: Chemistry. 2022 Apr 6;28(28):e202200376. doi: 10.1002/chem.202200376 (PMC9322665; doi:10.1002/chem.202200376)
Supplement: Supplementary file 1 — Supporting Information [file CHEM-28-0-s001.pdf]

Chemistry–A European Journal

Supporting Information

A Terphenyl Supported Dioxophosphorane Dimer: the Light Congener of Lawesson's and Woollins' Reagents

Laura E. English, Aleksandra Pajak, Claire L. McMullin, John P. Lowe, Mary F. Mahon, and David J. Liptrot*

Section 1: Experimental 2
General Considerations and Starting Materials 2
Synthesis of compounds **1**-**3** 3
NMR scale thermolysis of compound **1** 4
NMR scale reaction of compound **2** with DMAP 4
NMR scale reaction of compound **1** with DMAP 4
NMR scale thermal stability testing of compound **3** 4
NMR scale reaction of compound **1** with 20 mol% DMAP 4
NMR scale reaction of compound **1** with Pyridine 4
NMR scale reaction of compound **3** with $B(C_6F_5)_3$ 4
Section 2: X-Ray Crystallography 5
Table S1: Crystal data and structure refinement details 6
Figure S1: ORTEP representation of compound **1** 7
Section 3: Differential Scanning Calorimetry 8
Figure S2: Plot of DSC trace for compound **1** 8
Section 4: In Situ Reaction Monitoring by NMR Spectroscopy 9
Figures S3-S4: NMR scale thermolysis of compound **1** 9
Figures S5-S6: NMR scale reaction of compound **2** with DMAP 10
Figures S7-S8: NMR scale reaction of compound **1** with DMAP 11
Figures S9-S10: NMR scale thermal stability testing of compound **3** 12
Figures S11-S12: NMR scale reaction of compound **1** with 20 mol% DMAP 13
Figures S13-S14: NMR scale reaction of compound **1** with pyridine 14
Figures S15-S17: NMR scale reaction of compound **3** with $B(C_6F_5)_3$ 15
Section 5: Variable Temperature NMR Spectroscopy 17
Figures S18-S19: ^{31}P NMR spectra of compound **2** at varied temperature 17
Section 6: Diffusion Ordered Spectroscopy 18
^{1}H NMR DOSY data for compound **2** 19
Section 7: Computational Details and Methodology 20
Table S2: Relative energies for computed structures 20
Cartesian coordinates and computed energies 21
Section 8: NMR Spectra of Compounds 1-3 23
Figures S20-S22: NMR spectra of compound **1** 23
Figures S23-S25: NMR spectra of compound **2** 25
Figures S26-S28: NMR spectra of compound **3** 27
Section 9: IR Data for Compounds 2 and 3 29
Figure S29: IR spectrum of compound **2** 29
Figure S30: IR spectrum of compound **3** 29
Section 9: References 30

Section 1: Experimental

General Considerations and Starting Materials

Unless otherwise stated reactions were carried out under argon atmosphere using standard Schlenk line and glovebox techniques. NMR experiments using air-sensitive compounds were conducted in J. Young's tap NMR tubes prepared and sealed in a glovebox under argon. Toluene and hexane were purified using an MBraun Solvent Purification System and stored over 4 Å molecular sieves. THF and Et_2O were dried over sodium/benzophenone, then distilled and stored over 4 Å molecular sieves. Other non-deuterated solvents were used as supplied. d_6-benzene and d_8-toluene were dried over a potassium mirror prior to vacuum transfer into a sealed ampoule and stored in the glove box under argon. *d*-chloroform was used as received. All NMR data were acquired at 298 K on an Agilent ProPulse instrument for ^1H (500 MHz), ^{13}C (126 MHz) and ^{31}P (202 MHz), a Bruker AV400 instrument for ^1H (400 MHz) and ^{31}P (161 MHz), a Bruker AV300 spectrometer for ^1H (300 MHz) and ^{31}P (122 MHz), and a Bruker 500 MHz Advance II+ spectrometer for ^1H (500 MHz) and ^{31}P (202 MHz). ^1H and ^{13}C NMR spectra were referenced using residual solvent resonances. Mass spectrometry was performed using a Bruker MicrOTOF Electrospray Time-Of-Flight Mass Spectrometer coupled to an Agilent High Performance Liquid Chromatography unit. Differential Scanning Calorimetry was performed using a TA Instruments DSC Q20 Differential Scanning Calorimeter. Elemental analyses were performed by Elemental Microanalysis Ltd., Okehampton, Devon, U.K. IR was performed using a Thermo Scientific Nicolet™ iS™ 5 with an iD7 ATR Accessory. Commercially available reagents were purchased from relevant suppliers and used as supplied apart from 2-chloro-1,3,2-dioxaphospholane, which was distilled prior to use, DMAP, which was dried over 4 Å molecular sieves as an ethereal solution, pyridine, which was dried over CaH_2 then distilled, and $B(C_6F_5)_3$ which was purified by sublimation. (2,2'',6,6''-tetraisopropyl-[1,1':3',1''-terphenyl]-2'-yl)lithium was synthesised according to a literature procedure.[1]

Synthesis of compounds 1-3

Synthesis of compound 1, $Ar^{iPr_4}P(OCH_2)_2$: (2,2'',6,6''-tetraisopropyl-[1,1':3',1''-terphenyl]-2'-yl)lithium (1.799 g, 1 Eq, 4.447 mmol) was dissolved in ~30 mL Et_2O and added dropwise over 1.5 hours to a solution of 2-chloro-1,3,2-dioxaphospholane (592 mg, 416 µL, 1.05 Eq, 4.68 mmol) in ~30 mL Et_2O at 0 °C. This resulted in a white suspension which was allowed to warm to room temperature and stirred overnight. The solvent was removed from the reaction mixture *in vacuo* and the resulting solid extracted into hexane before filtering. The solvent was removed from the resulting colourless solution *in vacuo* to produce a beige powder. Yield: 1.75 g, 80%. Single crystals suitable for X-ray diffraction were grown from Et_2O at -35 °C. ^1H NMR (300 MHz, d_6-benzene) δ 7.35-7.30 (m, 2H, Ar*H*), 7.22 – 7.17 (m, 4H, Ar*H*), 7.10 – 7.03 (m, 3H, Ar*H*), 3.12 – 2.94 (m, 4H, P(OC*H*$_2$)$_2$), 2.85 (hept, $^3J_{H-H}$ = 6.9 Hz, 4H, C*H*(CH$_3$)$_2$), 1.36 (d, $^3J_{H-H}$ = 6.9 Hz, 12H, CH(C*H*$_3$)$_2$), 1.08 (d, $^3J_{H-H}$ = 6.9 Hz, 12H, CH(C*H*3)2). ^{13}C NMR (126 MHz, d_6-benzene) δ 147.2 (s, Ar*C*), 144.0 (d, $^1J_{C-P}$ = 17.7 Hz, Ar*C*), 138.6 (d, $^3J_{C-P}$ = 3.7 Hz, Ar*C*), 130.9 (s, Ar*C*), 128.9 (s, Ar*C*), 128.7 (s, Ar*C*), 122.8 (s, Ar*C*), 64.8 (d, $^2J_{C-P}$ = 8.2, P(O*C*H$_2$)$_2$), 31.6 (s, CH(*C*H$_3$)$_2$), 25.9 (s, *C*H(CH$_3$)$_2$), 22.7 (s, CH(*C*H$_3$)$_2$). ^{31}P NMR (122 MHz, d_6-benzene) δ 172.7. MS (ESI) Expected for [$Ar^{iPr_4}P(OCH_2)_2$+H]: 489.2924, found: 489.2951 [M+H]+ (err [ppm] = -0.23).

Synthesis of compound 2, [Ar^{iPr_4}PO$_2$]$_2$: compound **1** (1.00 g, 1 Eq, 2.05 mmol) was dissolved in ~ 5 mL toluene and added to a J. Young's ampoule with pyridine (162 mg, 166 µL, 1 Eq, 2.05 mmol). The reaction vessel was then sealed and heated to 100 °C with stirring. After two days the reaction was allowed to cool to room temperature, the mixture filtered and the solid washed with acetone. Yield: 708 mg, 75%. ^{1}H NMR (500 MHz, *d*-chloroform) δ 7.42-7.39 (m, 2H, Ar*H*), 7.37-7.34 (m, 4H, Ar*H*), 7.01-6.99 (m, 12H, Ar*H*), 2.41 (hept, $^{3}J_{H-H}$ = 6.8 Hz, 8H, C*H*(CH$_3$)$_2$), 0.84 (d, $^{3}J_{H-H}$ = 6.8 Hz, 24H, CH(C*H*$_3$)$_2$), 0.81 (d, $^{3}J_{H-H}$ = 6.8 Hz, 24H, CH(C*H*$_3$)$_2$). ^{13}C NMR (126 MHz, *d*-chloroform) δ 147.6 (s, Ar*C*), 143.7 (m, Ar*C*), 136.5 (m, Ar*C*), 131.6-131.5 (m, Ar*C*), 130.9 (s, Ar*C*), 129.7 (s, Ar*C*), 123.8 (s, Ar*C*), 31.3 (s, CH(*C*H$_3$)$_2$), 26.0 (s, *C*H(CH$_3$)$_2$), 23.0 (s, CH(*C*H$_3$)$_2$). ^{31}P NMR (202 MHz, *d*-chloroform) δ 17.8. IR (neat): *v*/cm^{-1} = 460(m), 508(m), 541(s), 612(w), 698(m), 753(s), 799(vs), 864(s), 1021(mb), 1130(m), 1182(mb), 1286(m), 1361(w), 1444(mb), 1566(w), 1688(w), 2866(w), 2958(m), 3063(w), 3309(wb), 3377(wb). MS (ESI) Expected for [(Ar^{iPr_4}PO$_2$)$_2$+Na]+: 943.4962, found: 943.4946 [M+Na]+ (err [ppm] = -0.39). Analysis calculated for (Ar^{iPr_4}PO$_2$)$_2$, $C_{60}H_{74}O_4P_2$ (MW = 920.50 g mol^{-1}): Expected C, 78.23; H, 8.10. Found C, 77.97; H, 8.00.

Synthesis of compound 3, Ar^{iPr_4}(DMAP)PO$_2$: compound **1** (1.00 g, 1 Eq, 2.05 mmol) and 4-dimethylaminopyridine (250 mg, 1 Eq, 2.05 mmol) were dissolved in ~10 mL THF in a vial in the glovebox. This was then left to sit at room temperature for 5 days, over which time a large amount of white precipitate formed. The solid was isolated by filtration and washed with THF before drying *in vacuo*. Single crystals suitable for X-ray diffraction were grown from slow evaporation of solution of **3** in iso-propanol at room temperature. Yield: 858 mg, 72%. ^{1}H NMR (500 MHz, *d*-chloroform) δ 7.62-7.58 (m, 2H), 7.30 – 7.21 (m, 3H), 7.12 (m, 1H), 7.11-7.09 (m, 5H), 6.24 – 6.20 (m, 2H), 3.11 (s, 6H), 2.66 (hept, $^{3}J_{H-H}$ = 6.8 Hz, 4H), 1.16 (d, $^{3}J_{H-H}$ = 6.8 Hz, 12H), 0.98 (d, $^{3}J_{H-H}$ = 6.8 Hz, 12H). ^{13}C NMR (126 MHz, *d*-chloroform) δ 156.4 (s, Ar*C*N), 146.9 (s, Ar*C*), 144.4 (s, Ar*C*), 143.4 (d, $^{3}J_{C-P}$ = 3.6 Hz, Ar*C*), 141.2 (d, $^{4}J_{C-P}$ = 3.2 Hz, Ar*C*), 139.4 (s, Ar*C*), 131.5 (d, $^{1}J_{C-P}$ = 14.0 Hz, Ar*C*), 128.5 (s, Ar*C*), 127.7 (s, Ar*C*), 122.3 (s, Ar*C*), 105.7 (d, $^{3}J_{C-P}$ = 3.8 Hz, Ar*C*), 40.3 (s, N(*C*H$_3$)$_2$, 31.0 (s, CH(*C*H$_3$)$_2$), 25.8 (s, *C*H(CH$_3$)$_2$), 22.9 (s, CH(*C*H$_3$)$_2$). ^{31}P NMR (202 MHz, *d*-chloroform) δ 6.2-6.3 (m). IR (neat): *v*/cm^{-1} = 457.58(m), 532.84(s), 706.51(w), 748.77(s), 831.17(m), 1024.9(vs), 1136.7(m), 1180.1(wb), 1231.5(w), 1291.1(m), 1398.3(w), 1442.1(w), 1559.6(w), 1623.7(m), 2865.5(w), 2957.2(wb), 3051.4(wb). MS (ESI) Expected for [Ar^{iPr_4}(DMAP)PO$_2$+H]: 583.3455, found: 583.3452 [M+H]+ (err [ppm] = 0.37). Analysis calculated for Ar^{iPr_4}(DMAP)PO$_2$·H$_2$O, $C_{37}H_{49}N_2O_3P$ (600.35 g mol^{-1}): Expected C, 73.97; H, 8.22; N, 4.66. Found: C, 74.44; H, 8.07; N, 4.90.

Note: Due to dissociation of DMAP from the adduct in solution, NMR spectra presented here are in the presence of a slight excess of DMAP, highlighted on the relevant NMR spectra.

***In Situ* NMR Scale Reactions**

NMR scale thermolysis of compound 1: compound **1** (50 mg, 1 Eq, 0.10 mmol) heated to 150 degrees without the addition of solvent in a J. Young's NMR tube. The reaction was monitored by ^{1}H and ^{31}P NMR spectroscopy after the addition of d_6-benzene to the NMR tube in the glovebox, running the NMR experiments and removal of the solvent *in vacuo* before returning to the heat. Once the reaction had proceeded to completion, the solvent was removed *in vacuo* and the solid washed with acetone to produce compound **2**. Single crystals suitable for X-ray diffraction were grown from the NMR sample in d_6-benzene at room temperature. Yield: 20.7 mg, 44%.

Note: The product of the reaction is only partially soluble in d_6-benzene resulting in impurity signals appearing larger in the *in situ* NMR spectra than that of the product.

NMR Scale reaction of compound 2 with DMAP: compound **2** (20 mg, 1 Eq, 43 µmol) was suspended in a solution of DMAP (5.3 mg, 1 Eq, 43 µmol) in d_6-benzene and transferred to a J. Young's NMR tube. Initial ^{1}H and ^{31}P spectroscopic analyses were performed. The reaction was then left at room temperature for and monitored by NMR spectroscopy or heated to 60 °C for 5 days and monitored by NMR spectroscopy.

NMR scale reaction of compound 1 with DMAP: compound **1** (30.0 mg, 1 Eq, 61.4 µmol) and DMAP (7.50 mg, 1 Eq, 61.4 µmol) taken up in d_6-benzene to produce a colourless solution which was transferred to a J. Young's NMR tube. Initial ^{1}H and ^{31}P spectroscopic analyses were performed. The reaction was then left at room temperature for 8 days and monitored by NMR spectroscopy.

NMR scale thermal stability testing of compound 3: compound **3** (25 mg, 1 Eq, 43 µmol) was suspended in d_6-benzene and the suspension transferred to a J. Young's NMR tube. The suspension was then heated to 80 °C for 3 days and ^{1}H and ^{31}P NMR spectroscopic analyses performed.

NMR scale reaction of compound 1 with 20 mol% DMAP: compound **1** (60.0 mg, 1 Eq, 123 µmol) and DMAP (3.00 mg, 0.2 Eq, 24.6 µmol) taken up in d_6-benzene to produce a colourless solution which was transferred to a J. Young's NMR tube. Initial ^{1}H and ^{31}P NMR spectroscopic analyses were performed, the reaction was heated to 100 °C and monitored by NMR spectroscopy.

NMR scale reaction of compound 1 with pyridine: compound **1** (50 mg, 1 Eq, 0.10 mmol) and pyridine (8.1 mg, 8.3 µL, 1 Eq, 0.10 mmol) were dissolved in d_6-benzene and transferred to a J. Young's NMR tube. Initial ^{1}H and ^{31}P NMR spectroscopic analyses were performed, the reaction was heated to 100 °C and monitored by NMR spectroscopy.

NMR scale reaction of compound 3 with $B(C_6F_5)_3$: d_6-benzene was added to $B(C_6F_5)_3$ (22 mg, 1 Eq, 43 µmol) and compound **3** (25 mg, 1 Eq, 43 µmol) producing a white suspension which was transferred to a J. Young's NMR tube. ^{1}H, ^{31}P and ^{19}F NMR spectroscopic analyses were performed.

Note: Poor shimming due to insolubility of components led to a very broad ^{1}H NMR spectrum.

Section 2: X-Ray Crystallography

Data for compounds **1-3** were collected on a RIGAKU SuperNova diffractometer with a Cu source (λ = 1.54184). The crystals were kept at 150.00(10) K during data collection. Using Olex2,[2] the structures were solved with the ShelXT[3] structure solution program using Intrinsic Phasing and refined with the ShelXL[4] refinement package using Least Squares minimisation.

The asymmetric unit in the structure of **1** comprises 2 phosphine molecules. Electron-density within bonding distance of P1 has been modelled as 20% of an oxygen atom (O3) which would suggest that the crystal as a whole contains 10% of a phosphine oxide. The P=O distance was refined to a distance restraint of 1.45(1) Å, it converged to a value of 1.375(5) Å.

The asymmetric unit in **2** is constituted by half of a centrosymmetric dimer.

The asymmetric unit in the structure of **3** contains one molecule of the main feature and two molecules of iso-propanol. The hydroxy groups in the latter are involved in hydrogen bonding.

**Table S1**: Crystal Data and Structure Refinement Details			
Identification code	**1**	**2**	**3**
Empirical formula	$C_{64}H_{82}O_{4.2}P_2$	$C_{30}H_{37}O_2P$	$C_{43}H_{63}N_2O_4P$
Formula weight	980.43	460.56	702.92
Crystal system	monoclinic	monoclinic	monoclinic
Space group	$P2_1/c$	$C2/c$	$P2_1/n$
a/ Å	17.9410(2)	21.5815(2)	10.24832(5)
b/ Å	9.34471(11)	14.9095(1)	16.85928(7)
c/ Å	33.5385(4)	16.1160(1)	24.46539(12)
α/ °	90	90	90
β/ °	94.6640(11)	95.560(1)	96.6178(4)
γ/ °	90	90	90
U/ Å^3	5604.23(11)	5161.24(7)	4198.95(3)
Z	4	8	4
ρ_{calc}/ g cm^{-3}	1.162	1.185	1.112
μ/ mm^{-1}	1.059	1.117	0.891
F(000)	2118.0	1984.0	1528.0
Crystal size/ mm^3	0.301 × 0.222 × 0.12	0.122 × 0.115 × 0.097	274 × 0.207 × 0.14
2θ range for data collection/°	CuKα (λ = 1.54184)	Cu Kα (λ = 1.54184)	Cu Kα (λ = 1.54184)
Index ranges	5.288 to 146.658	7.218 to 145.872	7.276 to 147.158
Reflections collected	−19 ≤ h ≤ 22,<br>−11 ≤ k ≤ 9,<br>−41 ≤ l ≤ 41	−26 ≤ h ≤ 23,<br>−17 ≤ k ≤ 18,<br>−19 ≤ l ≤ 19	−12 ≤ h ≤ 12,<br>−20 ≤ k ≤ 14,<br>−30 ≤ l ≤ 30
Independent reflections, R_{int}	39074	17156	57865
Data/restraints/parameters	11098, 0.0254	5101, 0.0162	8430, 0.0205
Goodness–of–fit on F^2	11098/1/656	5101/0/306	8430/2/473
Final R1, wR2 [I>=2σ(I)]	1.026	1.036	1.024
Final R1, wR2 [all data]	0.0457, 0.1113	0.0342, 0.0895	0.0394, 0.1036
Largest diff. peak/hole/ e Å^{-3}	0.0487, 0.1137	0.0356, 0.0907	0.0409, 0.1049
Identification code	1.41/−0.64	0.29/−0.40	0.31/−0.36

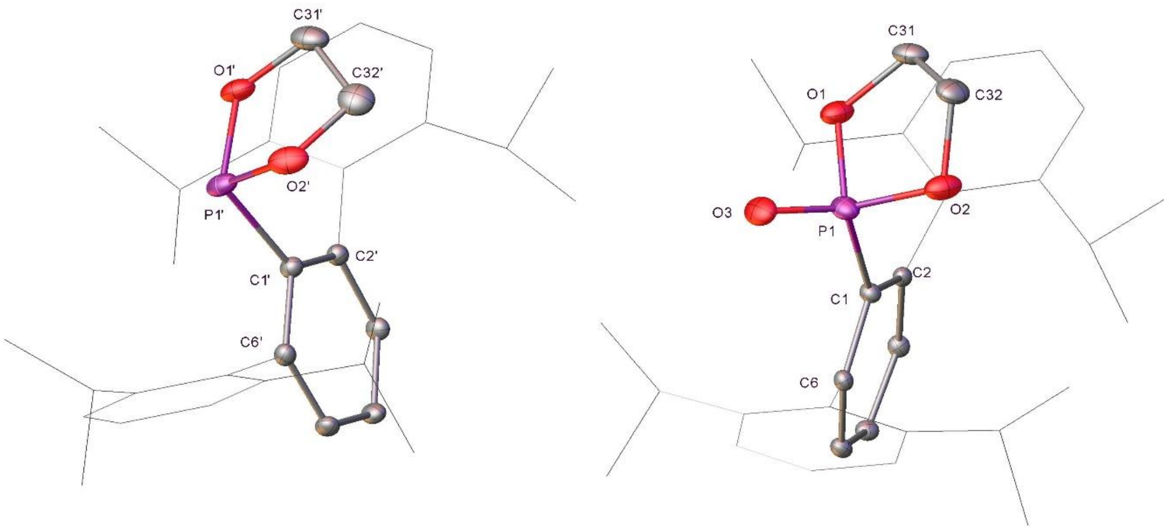

Figure S1: ORTEP representation of compound **1** (30% probability elipsoids). Hydrogen atoms are omitted for clarity.

Section 3: Differential Scanning Calorimetry

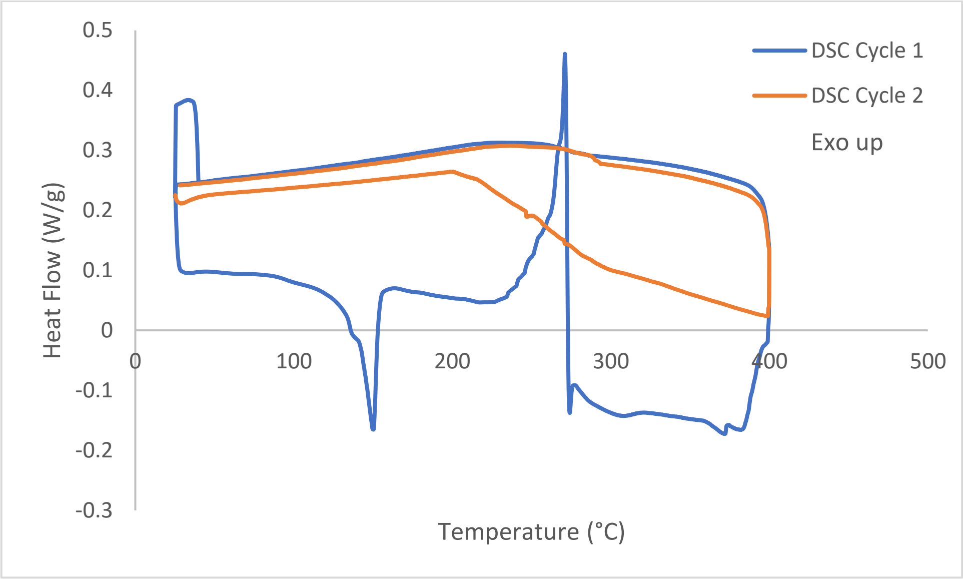

Figure S2: Plot of DSC trace for compound **1** collected over two heating and cooling cycles from 25 °C to 400 °C.

Section 4: *In Situ* Reaction Monitoring by NMR Spectroscopy

NMR scale thermolysis of compound 1:

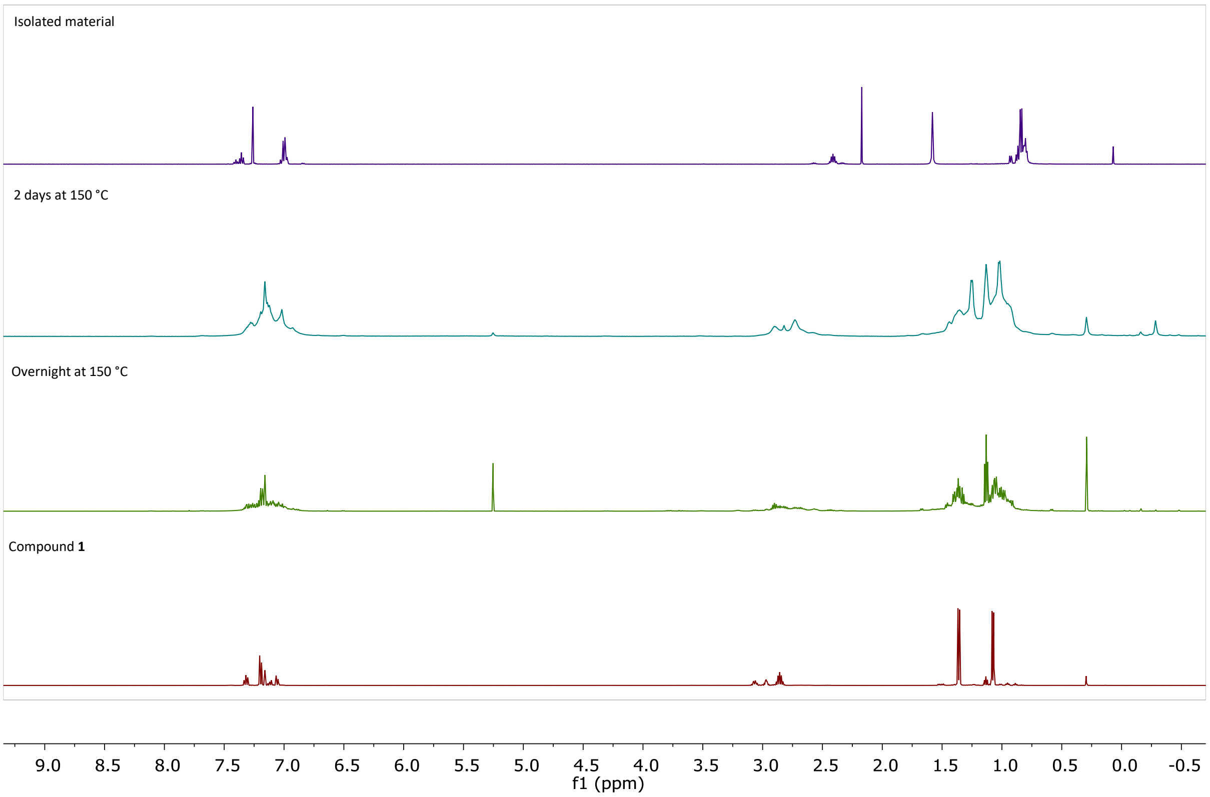

Figure S3: Stacked ^{1}H NMR spectra of thermolysis of compound **1** having dissolved residues in d_6-benzene, apart from isolated material NMR spectrum which is in *d*-chloroform.

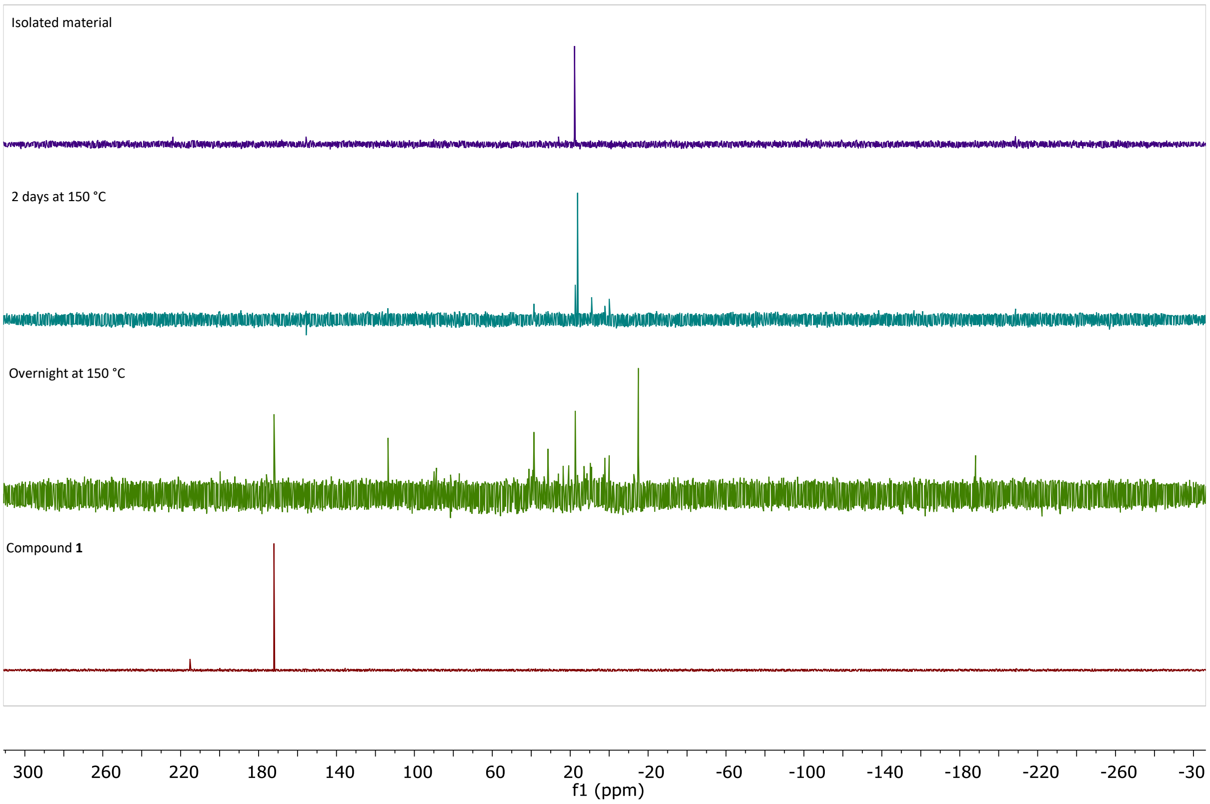

Figure S4: Stacked ^{31}P NMR spectra of thermolysis of compound **1** having dissolved residues in d_6-benzene, apart from isolated material NMR spectrum which is in *d*-chloroform.

NMR scale reaction of compound 2 with DMAP

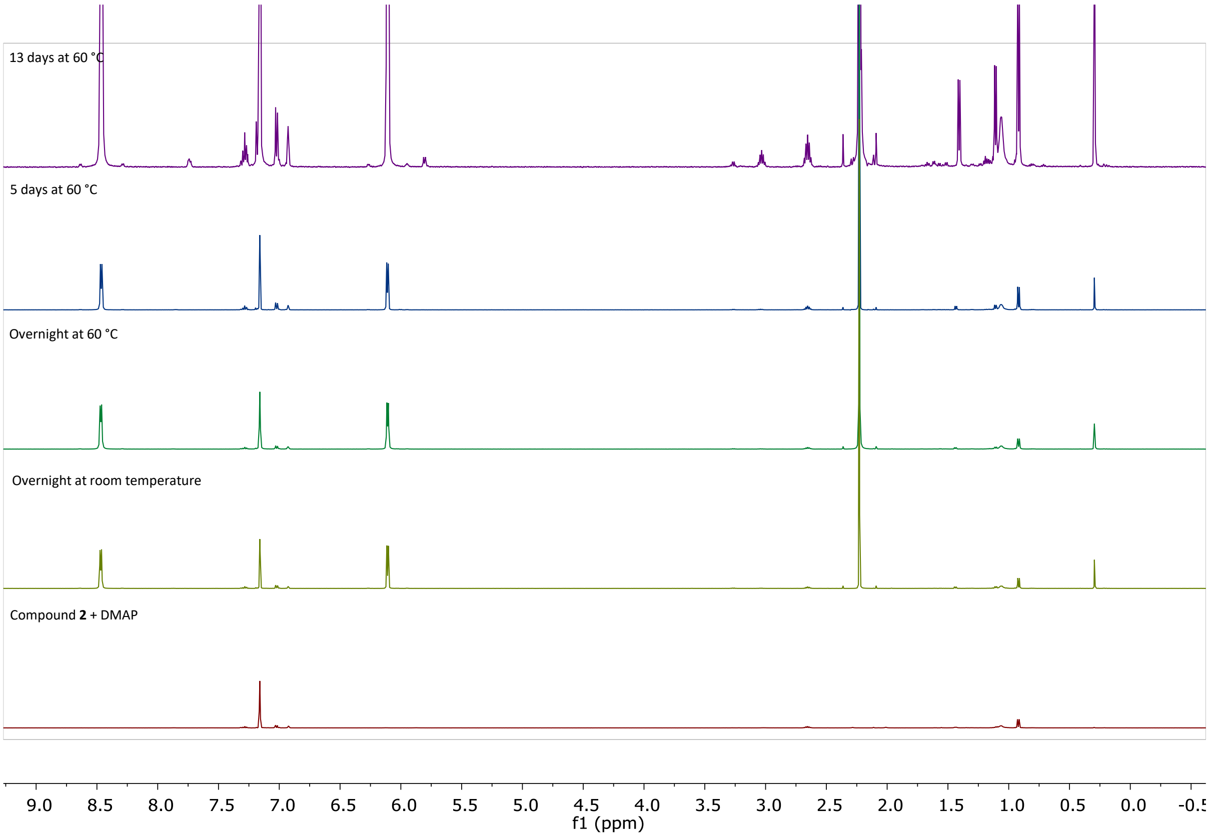

Figure S5: Stacked ^{1}H NMR spectra of reaction of compound **2** with 1 equiv. DMAP in d_6-benzene.

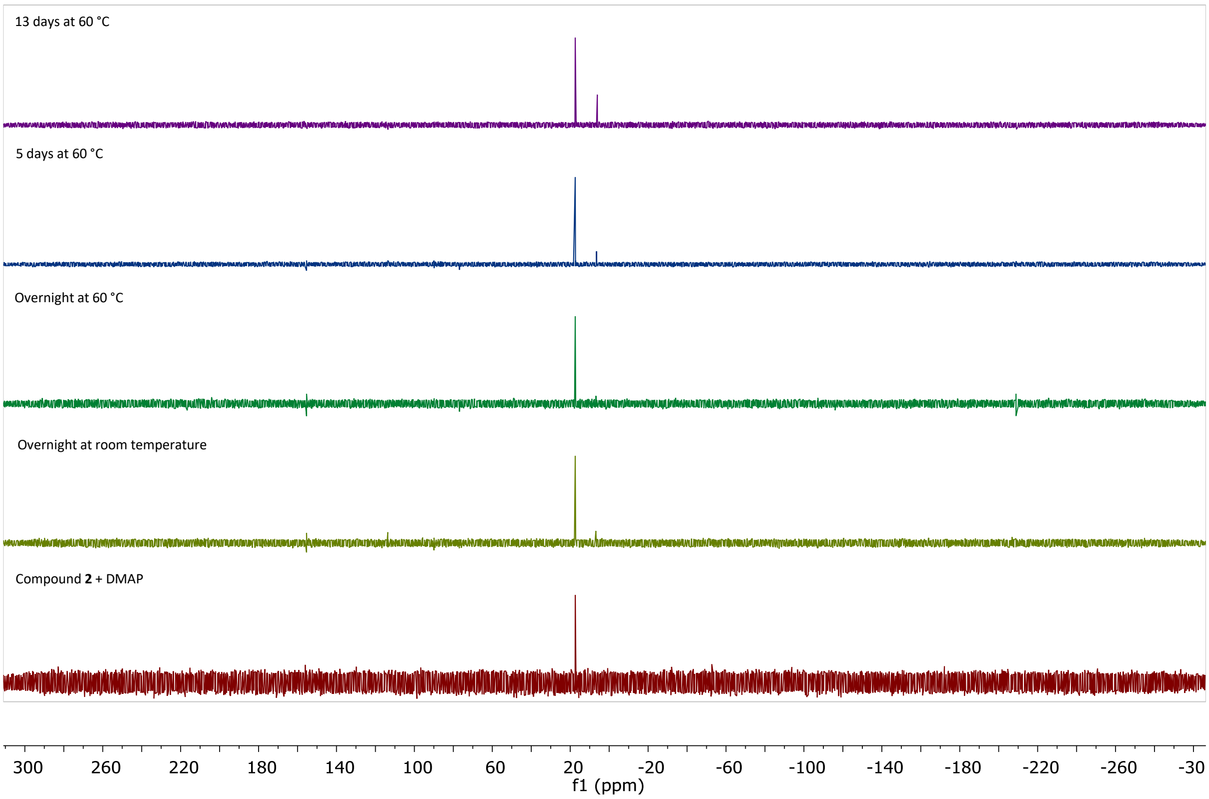

Figure S6: Stacked ^{31}P NMR spectra of reaction of compound **2** with 1 equiv. DMAP in d_6-benzene.

NMR scale reaction of compound 1 with DMAP

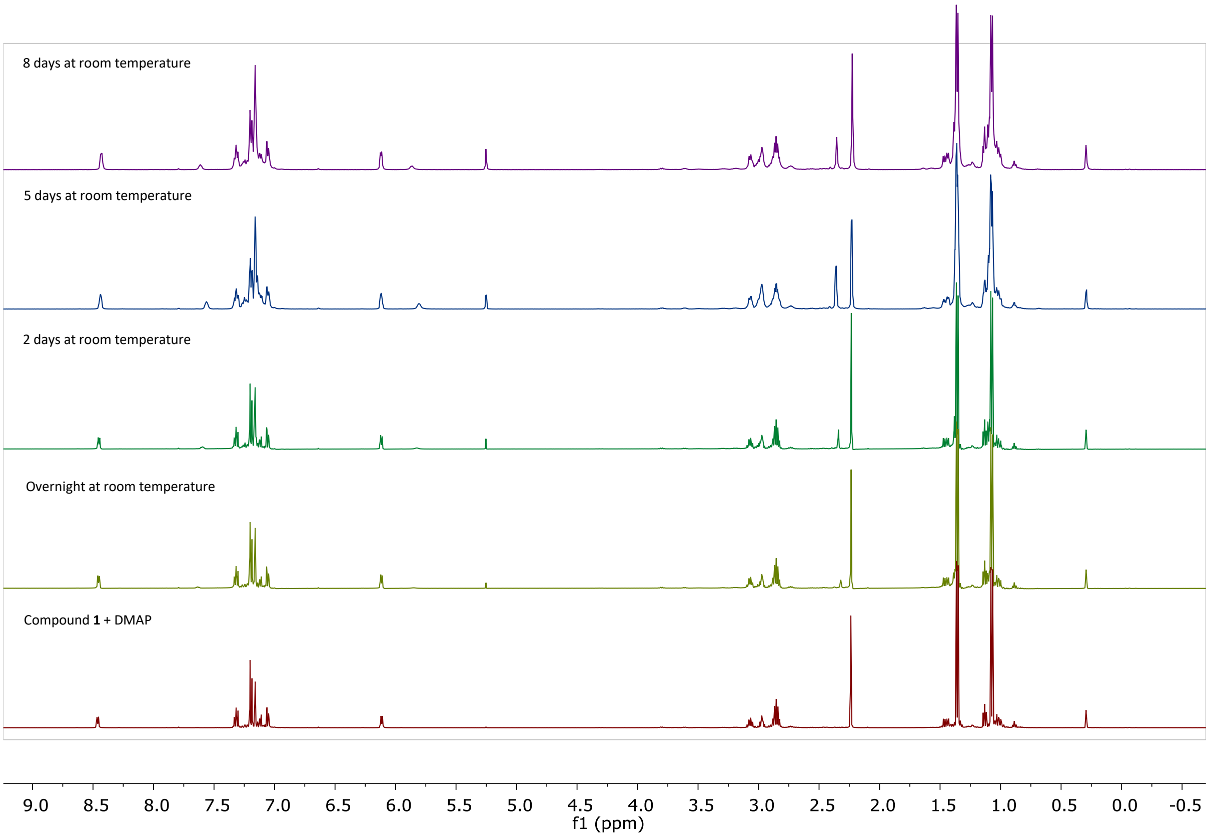

Figure S7: Stacked ^{1}H NMR spectra of reaction of compound **1** with 1 equiv. DMAP in d_6-benzene.

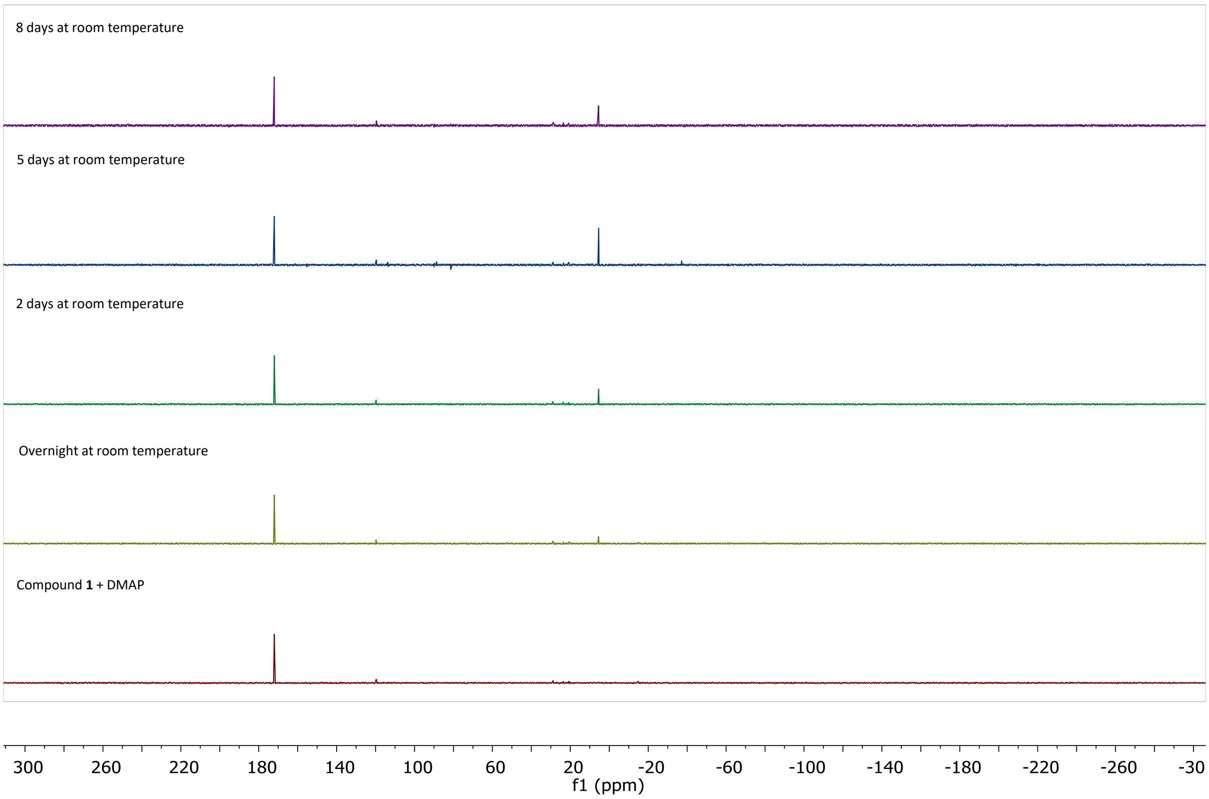

Figure S8: Stacked ^{31}P NMR spectra of reaction of compound **1** with 1 equiv. DMAP in d_6-benzene.

NMR scale thermal stability testing of compound 3

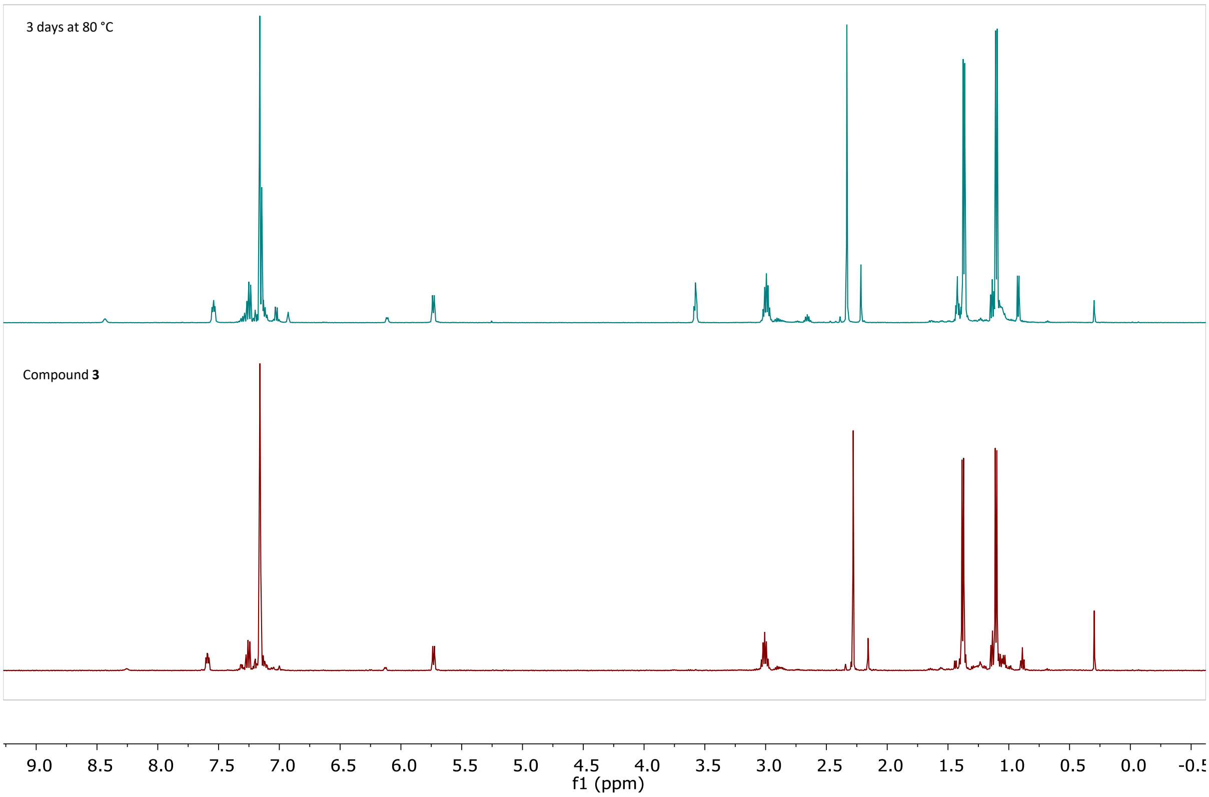

Figure S9: Stacked ^{1}H NMR spectra of thermal stability testing of compound **3** in d_6-benzene.

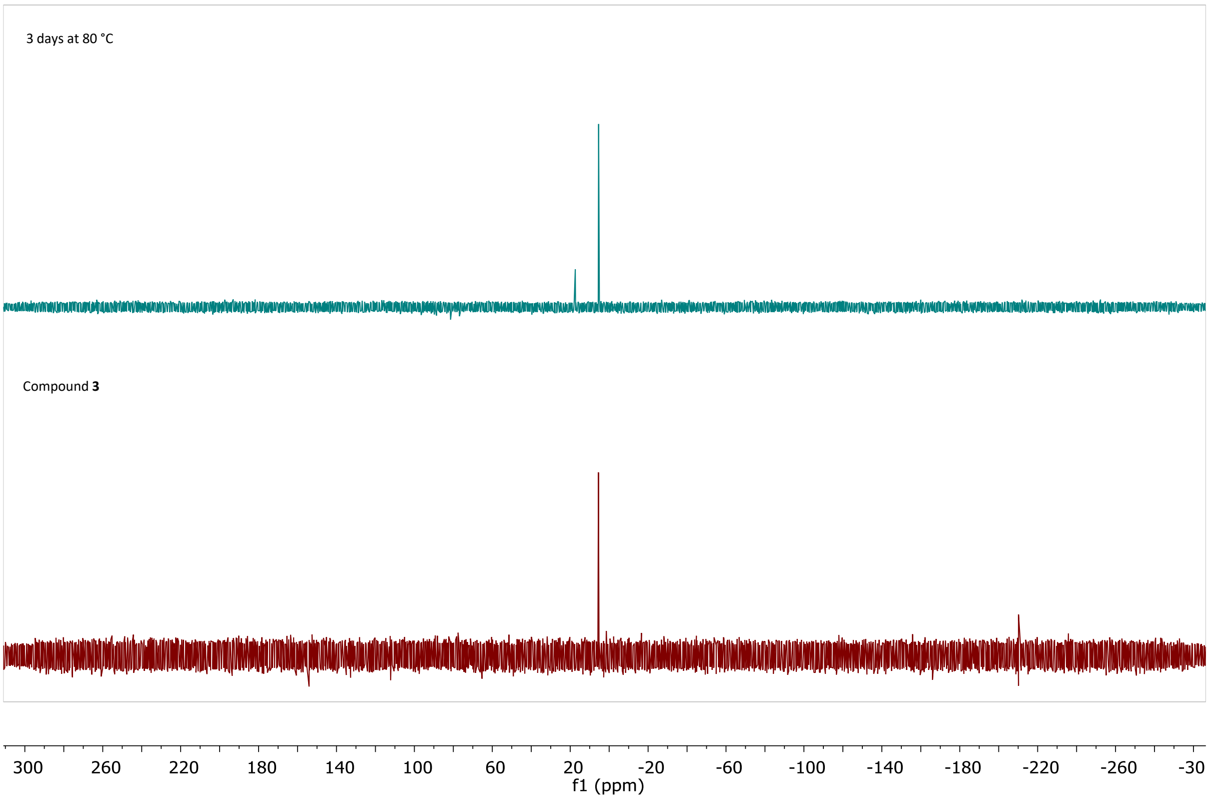

Figure S10: Stacked ^{31}P NMR spectra of thermal stability testing of compound **3** in d_6-benzene.

NMR scale reaction of compound 1 with 20 mol% DMAP

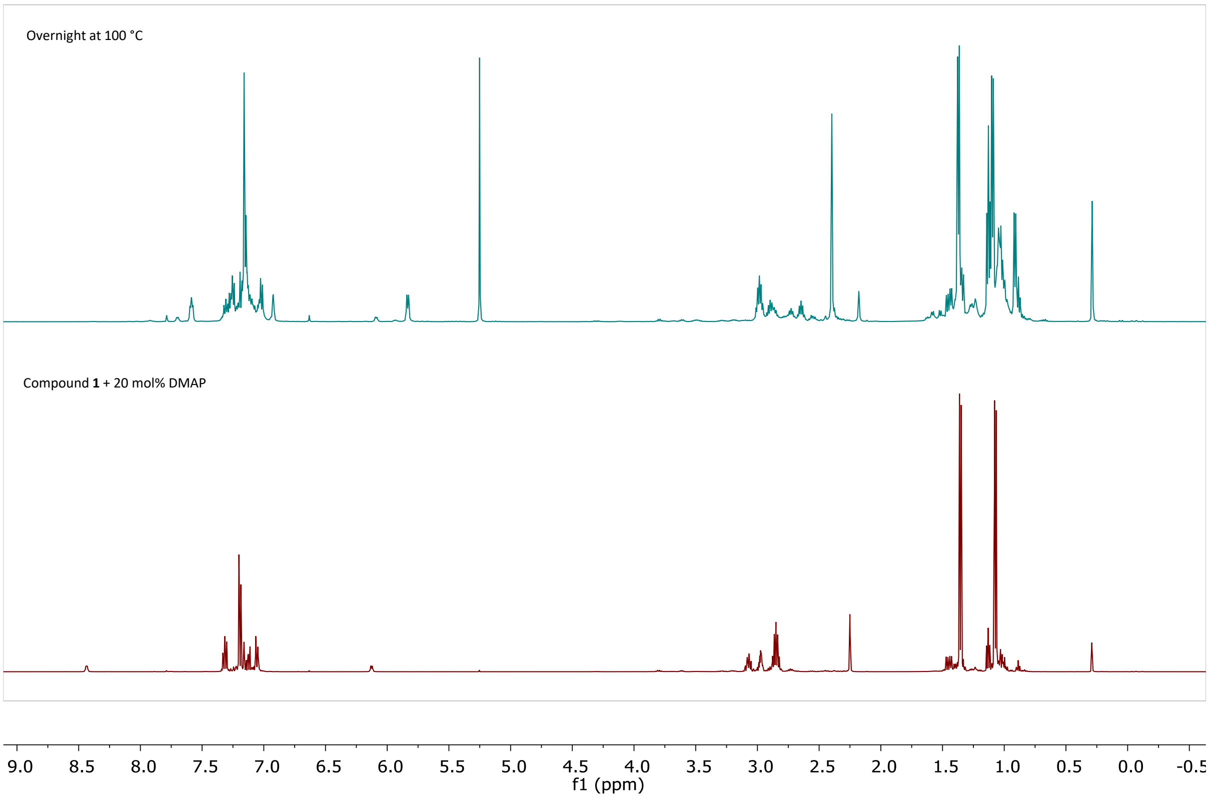

Figure S11: Stacked ^{1}H NMR spectra of reaction of compound **1** with 0.2 equiv. DMAP in d_6-benzene.

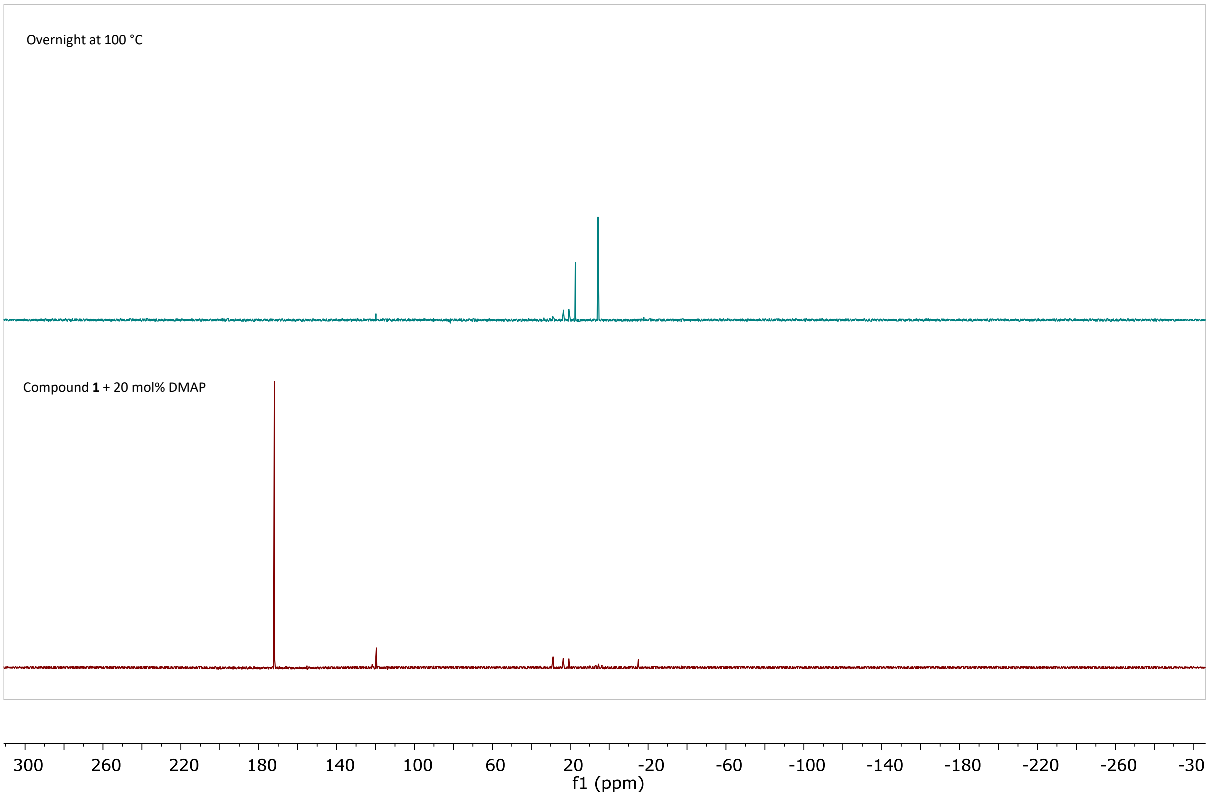

Figure S12: Stacked ^{31}P NMR spectra of reaction of compound **1** with 0.2 equiv. DMAP in d_6-benzene.

NMR scale reaction of compound 1 with pyridine

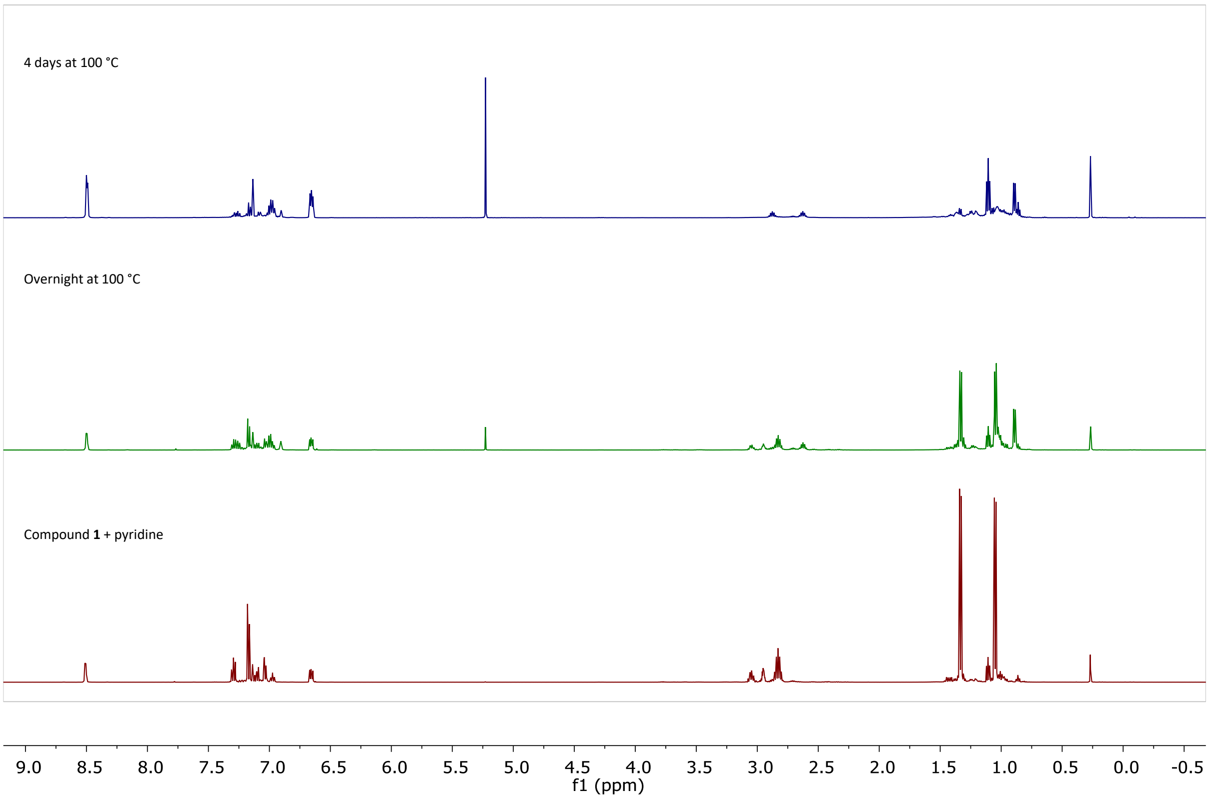

Figure S13: Stacked ^{1}H NMR spectra of reaction of compound **1** with 1 equiv. pyridine in d_6-benzene.

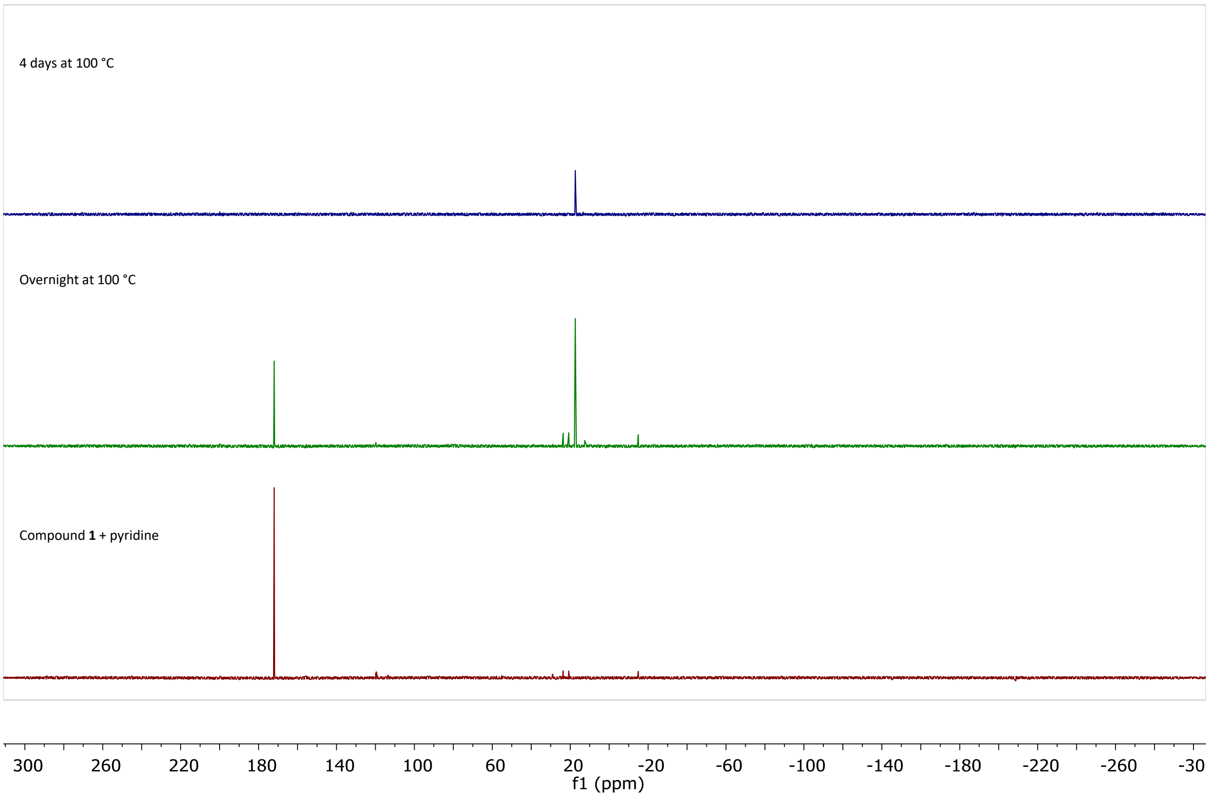

Figure S14: Stacked ^{31}P NMR spectra of reaction of compound **1** with 1 equiv. pyridine in d_6-benzene.

NMR scale reaction of compound 3 with $B(C_6F_5)_3$

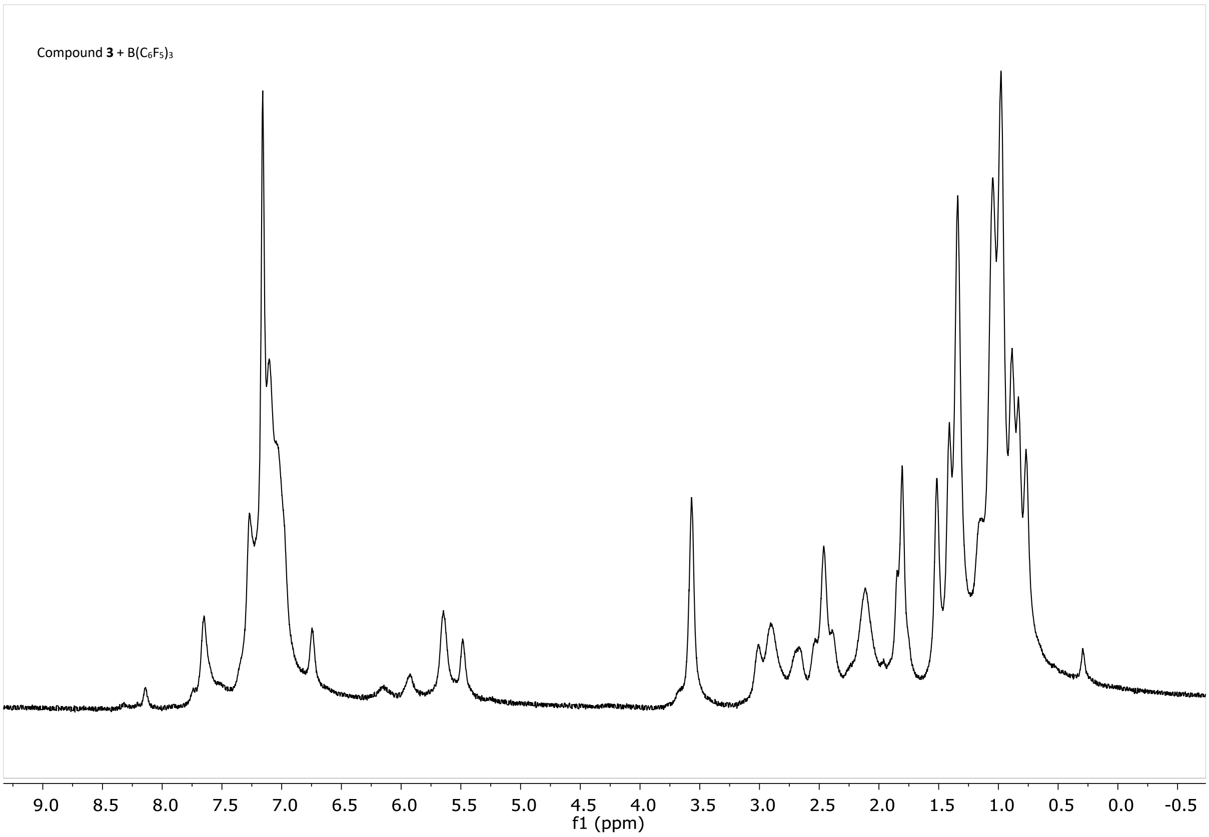

Figure S15: ^{1}H NMR spectrum of reaction of compound **3** with 1 equiv. $B(C_6F_5)_3$ in d_6-benzene.

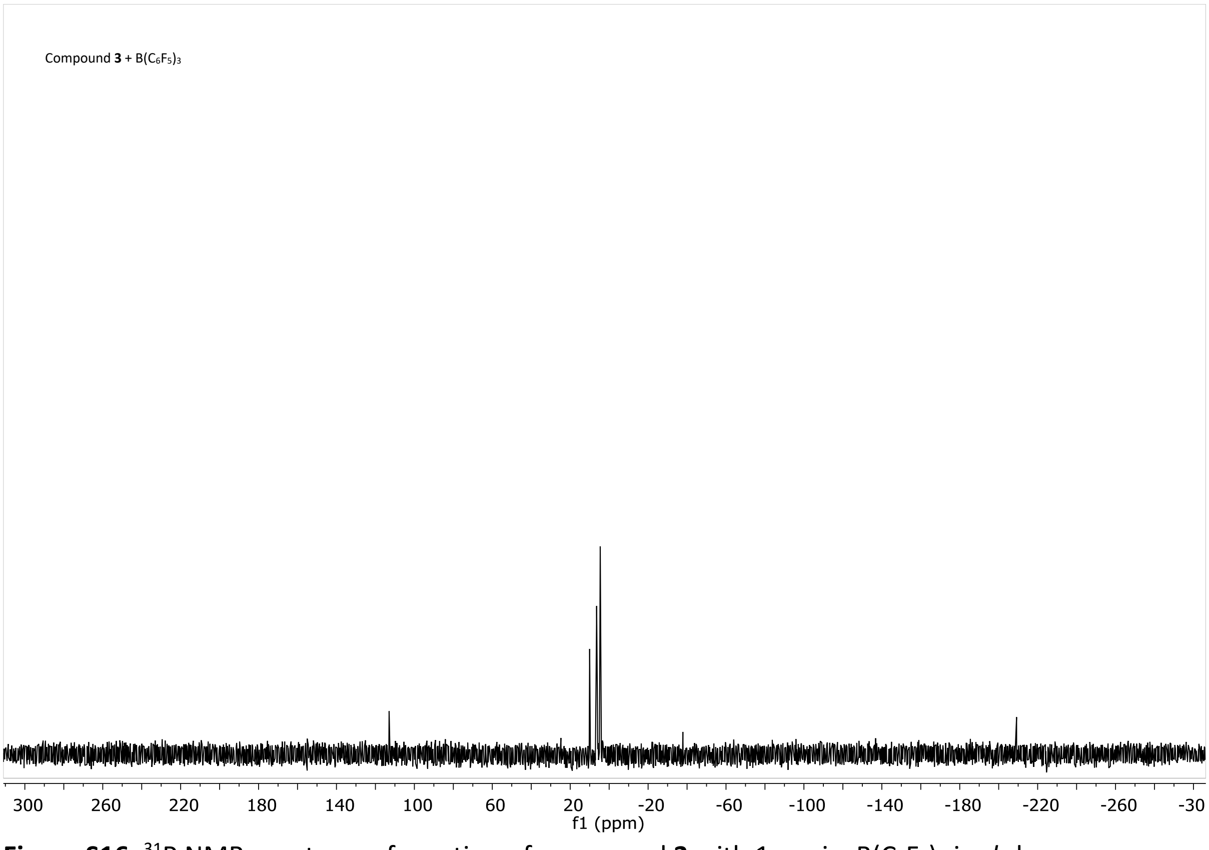

Figure S16: ^{31}P NMR spectrum of reaction of compound **3** with 1 equiv. $B(C_6F_5)_3$ in d_6-benzene.

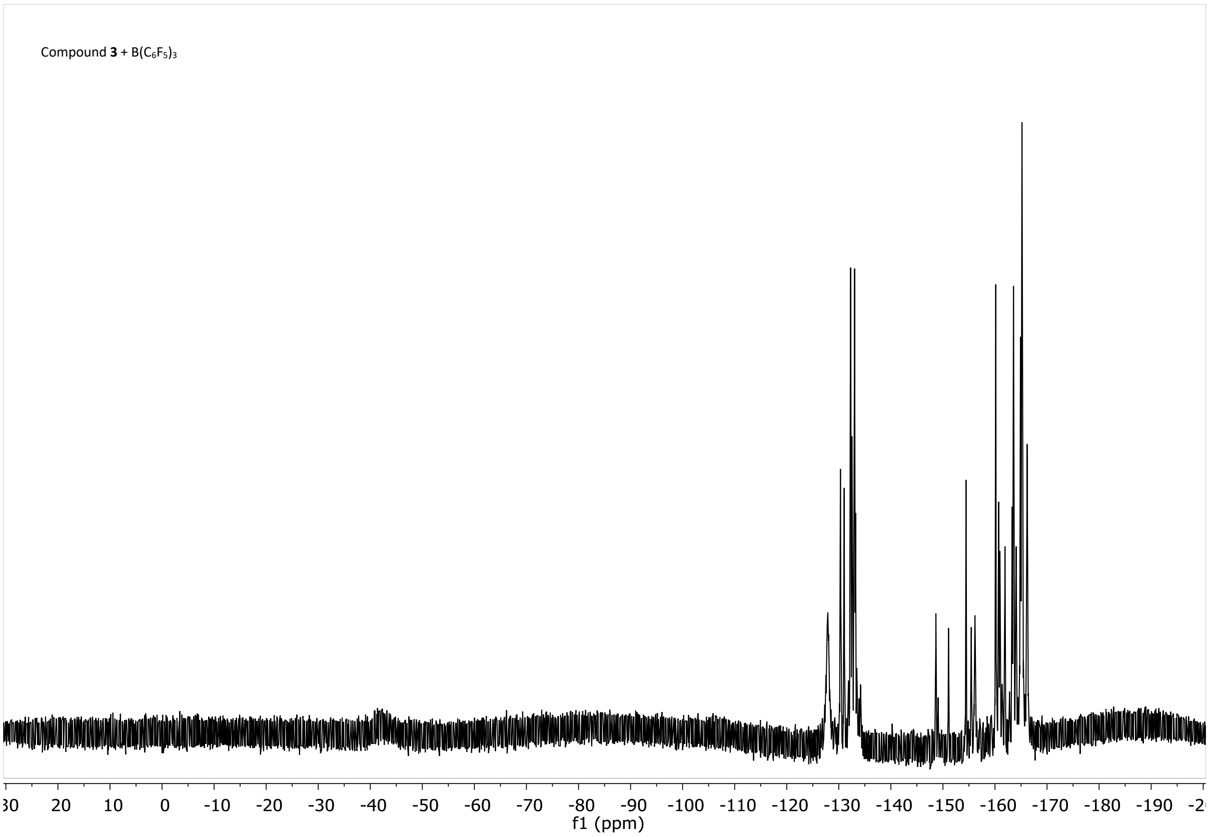

Figure S17: ^{19}F NMR spectrum of reaction of compound **3** with 1 equiv. $B(C_6F_5)_3$ in d_6-benzene.

Section 5: Variable Temperature NMR Spectroscopy

^{31}P NMR analysis was performed on a solution of compound **2** in d_8-toluene at 298 – 358 K.

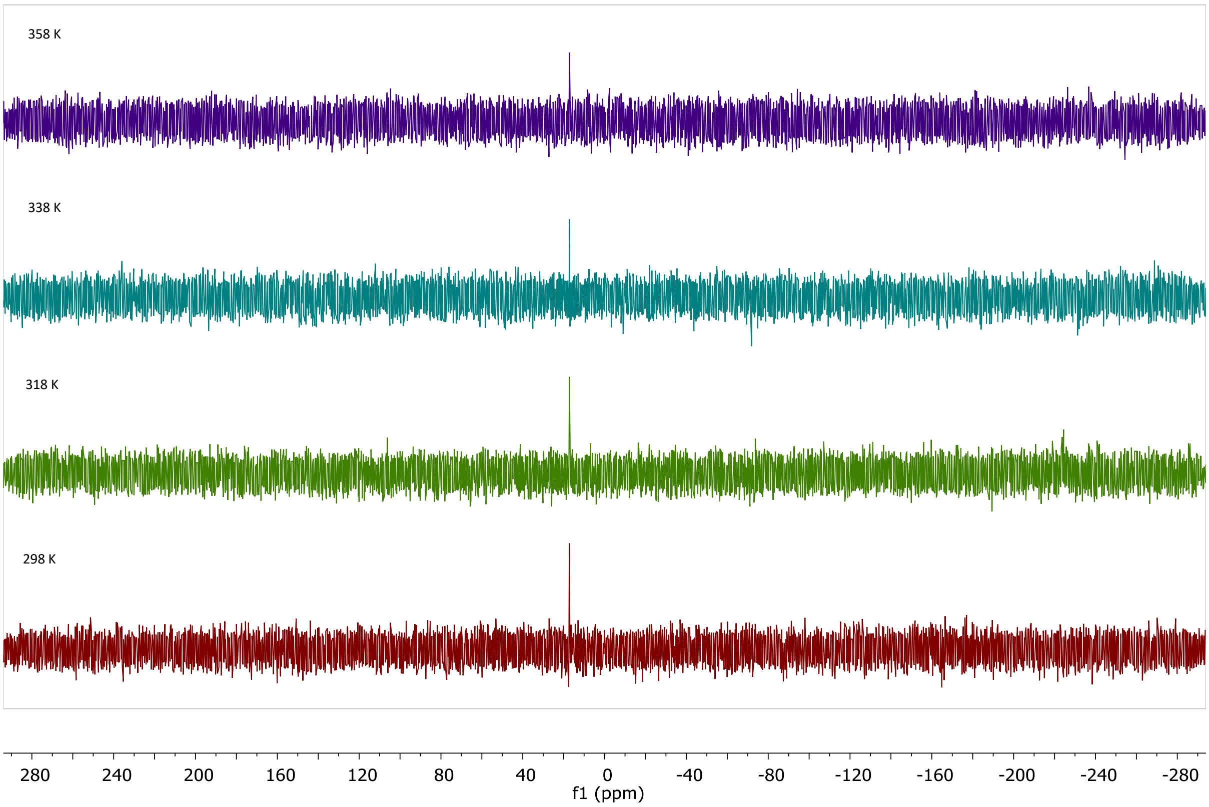

Figure S18: Stacked ^{31}P NMR Spectra of compound **2** at different temperatures.

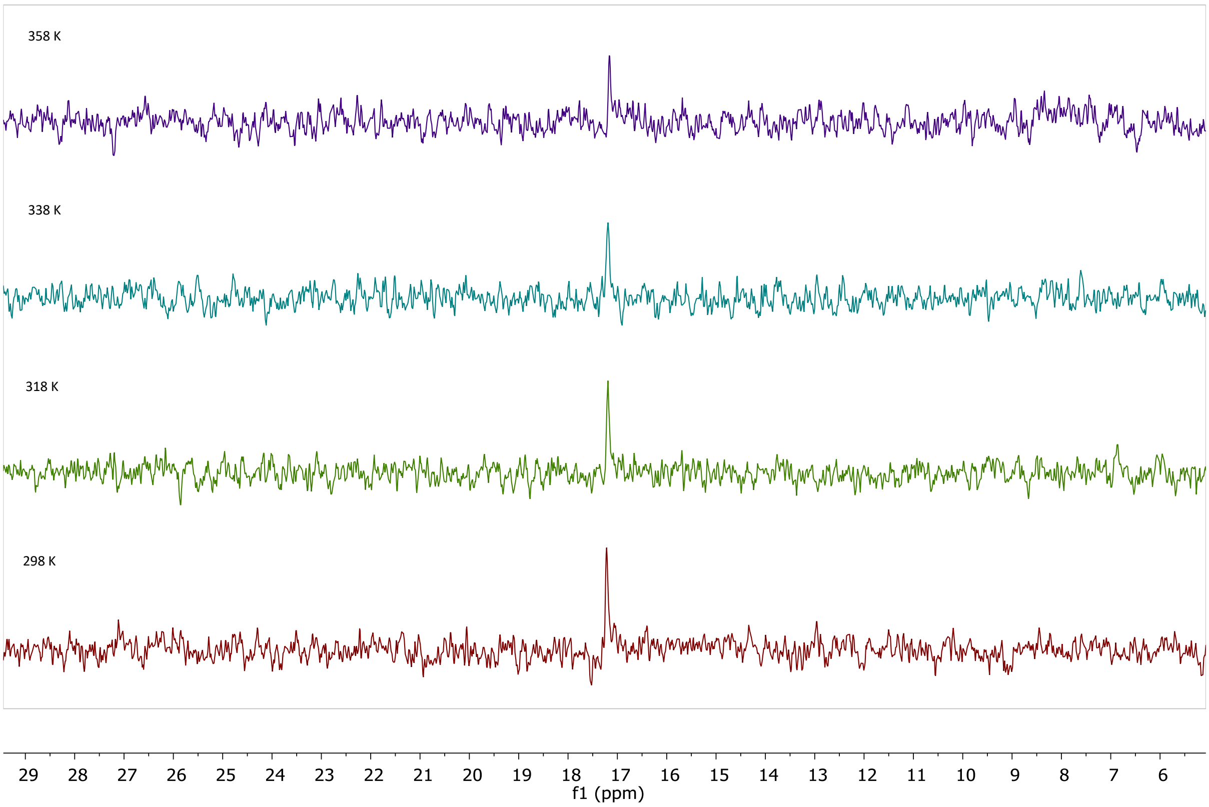

Figure S19: Stacked ^{31}P NMR Spectra of compound **2** at different temperatures, zoomed in to 5 – 29 ppm region.

Section 6: Diffusion Ordered Spectroscopy

DOSY was performed using ^{1}H resonances associated with the iso-propyl C*H* compound **2** in *d*-chloroform. The diffusion coefficient was converted to a hydrodynamic radius using the following equation:

$$D = \frac{kT}{6\pi\eta r_H}$$

Diffusion Coefficient / $\times 10^{-10}$ m^2s^{-1}	Hydrodynamic Radius / Å
6.63	6.14

Where D = diffusion coefficient, k = Boltzmann constant, T = absolute temperature, η = viscosity and r_H = hydrodynamic radius.

Calculated volumes were obtained from X-ray structures or computational .xyz files using the molinfo command in Olex2.[2] These were then converted into radii using the following equation:

$$r = \sqrt[3]{\frac{3V}{4\pi}}$$

	$Volume_{comp}$ / $Å^3$	$Radius_{comp}$ / Å	$Volume_{X\text{-}ray}$ / $Å^3$	$Radius_{X\text{-}ray}$ / Å
$Ar^{iPr_4}PO_2$	434.16	4.70	411.15	4.61
$[Ar^{iPr_4}PO_2]_2$	861.99	5.90	813.43	5.79

^{1}H NMR DOSY data for compound 2

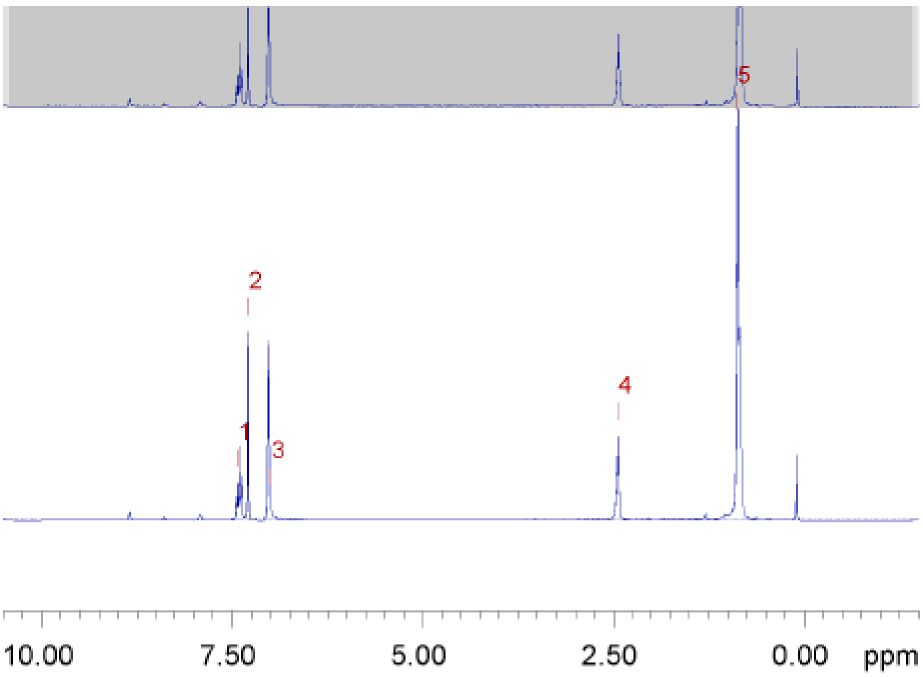

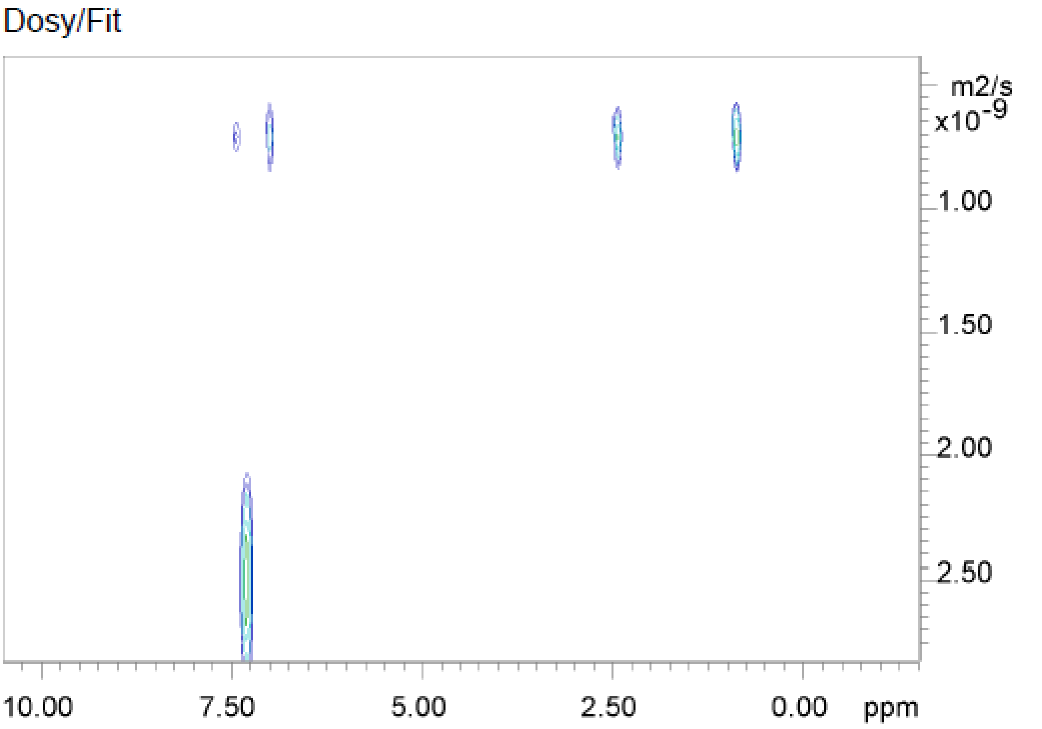

Peak	Chemical Shift / ppm	Diffusion Coefficient / x10^{-10} m^2s^{-1}
1	7.43	6.57
2	Omitted (*d*-chloroform)	-
3	6.99	7.08
4	2.44	6.63
5	0.88	6.64

Section 7: Computational Details and Methodology

DFT calculations were run with Gaussian 16 (C.01).[5] The P centres were described with the Stuttgart RECPs and associated basis sets,[6] whilst the 6-31G** basis set was used for all other atoms (BS1).[7] A polarization function was also added to P (ζ_d = 0.387). Initial BP86[8] optimizations were performed using the 'grid = ultrafine' option, with all stationary points being fully characterized via analytical frequency calculations as minima (all positive eigenvalues). Energies were recomputed with 6-311++G** basis sets for all atoms (BS2). Corrections for the effect of benzene (ε = 2.2706) solvent were run using the polarizable continuum model (PCM) and BS1.[9] Single-point dispersion corrections to the BP86 results employed Grimme's D3 parameter set with Becke-Johnson damping as implemented in Gaussian.[10]

Breakdown of Energy Contributions

The following tables detail the evolution of the relative energies as the successive corrections to the initial SCF energy are included. Terms used are:

**ΔE_{BS1}**	SCF energy computed with the BP86 functional with BS1
**ΔH_{BS1}**	Enthalpy at 0 K with BS1
**ΔG_{BS1}**	Free energy at 298.15 K and 1 atm with BS1
**$\Delta G_{BS1/bnz}$**	Free energy corrected for benzene solvent with BS1
**$\Delta G_{BS1/bnz+D3}$**	Free energy corrected for benzene and dispersion effects with BS1
**ΔE_{BS2}**	SCF energy computed with the BP86 functional with BS2
**ΔG_{bnz}**	Free energy corrected for basis set (BS2), dispersion effects and benzene solvent

In each case the final data used in the main article is highlighted in bold.

Table S2: Relative energies (kcal mol^{-1}) for computed structures. Data in bold are those used in the main text. All energies are quoted relative to $[Ar^{iPr_4}PO_2]_2$ at 0.0 kcal mol^{-1}.

	**ΔE_{BS1}**	**ΔH_{BS1}**	**ΔG_{BS1}**	**$\Delta G_{BS1/bnz}$**	**$\Delta G_{BS1/bnz+D3}$**	**ΔE_{BS2}**	**ΔG_{bnz}**
$[Ar^{iPr_4}PO_2]_2$	0.0	0.0	0.0	0.0	0.0	0.0	**0.0**
$Ar^{iPr_4}PO_2$	13.5	12.5	2.4	1.7	18.7	8.9	**14.2**

Cartesian Coordinates and Computed Energies [in Hartrees]

$[Ar^{iPr_4}PO_2]_2$ (Compound **2**)

```
SCF (BP86) Energy = -2645.10593230
Enthalpy 0K = -2643.942035
Enthalpy 298K = -2643.869224
Free Energy 298K = -2644.051662
Lowest Frequency = 11.1260 cm-1
Second Frequency = 18.2817 cm-1
SCF (Bnz) Energy = -2645.11140019
SCF (BP86-D3BJ) Energy = -
2645.50686460
SCF (BS2) Energy = -3315.40636522

P  -1.15160 -0.00041  0.49376
O   0.00047 -1.17264  0.00014
O  -1.51461 -0.00041  1.95947
C  -2.58804 -0.00116 -0.66121
C  -3.18511  1.23742 -1.03830
C  -4.33173  1.20563 -1.86112
H  -4.78829  2.15862 -2.14514
C  -4.89475 -0.00237 -2.28440
H  -5.78675 -0.00284 -2.91942
C  -4.33059 -1.20978 -1.86094
H  -4.78626 -2.16325 -2.14479
C  -3.18392 -1.24037 -1.03814
C  -2.73635  2.59863 -0.57343
C  -3.25205  3.11383  0.65114
C  -2.93442  4.44072  1.00476
H  -3.32674  4.85406  1.93990
C  -2.14344  5.24200  0.17429
H  -1.91249  6.27268  0.46526
C  -1.65819  4.72980 -1.03517
H  -1.04523  5.36469 -1.68268
C  -1.94718  3.41221 -1.43527
C  -2.73387 -2.60112 -0.57316
C  -1.94389 -3.41400 -1.43490
C  -1.65369 -4.73131 -1.03472
H  -1.04007 -5.36564 -1.68214
C  -2.13857 -5.24391  0.17473
H  -1.90669 -6.27437  0.46575
C  -2.93037 -4.44333  1.00509
H  -3.32238 -4.85699  1.94022
C  -3.24917 -3.11673  0.65140
C  -4.22028  2.31598  1.53170
H  -4.18919  1.26244  1.20397
C  -3.84230  2.33158  3.02705
H  -2.83834  1.90792  3.18175
H  -4.56238  1.72374  3.60218
H  -3.86605  3.35148  3.44963
C  -5.66709  2.82402  1.32238
H  -5.76225  3.88083  1.62869
H  -6.37837  2.23112  1.92400
H  -5.97194  2.75339  0.26449
C  -1.46495  2.90890 -2.79823
H  -1.56114  1.80855 -2.80318
C   0.01466  3.23884 -3.07512
H   0.17924  4.32488 -3.18873
H   0.33732  2.75841 -4.01463
H   0.66389  2.87941 -2.26372
C  -2.35862  3.46403 -3.93351
H  -3.41472  3.17322 -3.80675
H  -2.01741  3.08754 -4.91391
H  -2.31559  4.56738 -3.96010
C  -1.46209 -2.91032 -2.79788
H  -1.55925 -1.81006 -2.80287
C   0.01781 -3.23896 -3.07478
H   0.66675 -2.87883 -2.26346
H   0.33999 -2.75834 -4.01436
H   0.18338 -4.32486 -3.18826
C  -2.35527 -3.46630 -3.93314
H  -2.31127 -4.56961 -3.95967
H  -2.01438 -3.08956 -4.91355
H  -3.41162 -3.17641 -3.80640
C  -4.21811 -2.31972  1.53194
H  -4.18817 -1.26622  1.20399
C  -5.66442 -2.82929  1.32297
H  -5.96952 -2.75920  0.26511
H  -6.37621 -2.23698  1.92458
H  -5.75847 -3.88613  1.62953
C  -3.83983 -2.33465  3.02723
H  -3.86242 -3.35451  3.44996
H  -4.56045 -1.72748  3.60239
H  -2.83630 -1.90989  3.18167
P   1.15156  0.00048 -0.49372
O  -0.00051  1.17269 -0.00008
O   1.51453  0.00050 -1.95943
C   2.58803  0.00117  0.66121
C   3.18515 -1.23745  1.03812
C   4.33180 -1.20573  1.86090
H   4.78840 -2.15875  2.14477
C   4.89478  0.00223  2.28435
H   5.78680  0.00264  2.91935
C   4.33056  1.20967  1.86108
H   4.78620  2.16312  2.14507
C   3.18389  1.24034  1.03830
C   2.73641 -2.59864  0.57317
C   3.25220 -3.11381 -0.65138
C   2.93468 -4.44072 -1.00501
H   3.32709 -4.85404 -1.94014
C   2.14366 -5.24204 -0.17462
H   1.91278 -6.27273 -0.46560
C   1.65830 -4.72986  1.03481
H   1.04530 -5.36477  1.68226
C   1.94722 -3.41226  1.43495
C   2.73382  2.60112  0.57343
C   1.94394  3.41398  1.43529
C   1.65372  4.73130  1.03518
H   1.04019  5.36563  1.68270
C   2.13845  5.24393 -0.17431
H   1.90655  6.27440 -0.46528
C   2.93015  4.44337 -1.00478
H   3.32207  4.85706 -1.93995
C   3.24900  3.11677 -0.65116
C   4.22035 -2.31588 -1.53196
H   4.18938 -1.26239 -1.20404
C   3.84205 -2.33122 -3.02724
H   2.83810 -1.90745 -3.18167
H   4.56206 -1.72336 -3.60244
H   3.86563 -3.35107 -3.44995
C   5.66716 -2.82402 -1.32302
H   5.76223 -3.88078 -1.62955
H   6.37835 -2.23104 -1.92469
H   5.97225 -2.75359 -0.26519
C   1.46495 -2.90903  2.79792
```

```
H   1.56114 -1.80867  2.80293
C  -0.01467 -3.23897  3.07477
H  -0.17930 -4.32502  3.18816
H  -0.33730 -2.75871  4.01437
H  -0.66389 -2.87935  2.26344
C   2.35861 -3.46422  3.93317
H   3.41472 -3.17344  3.80640
H   2.01743 -3.08775  4.91359
H   2.31555 -4.56757  3.95973
C   1.46226  2.91026  2.79829
H   1.55950  1.81000  2.80327
C  -0.01765  3.23879  3.07529
H  -0.66661  2.87857  2.26402
H  -0.33972  2.75818  4.01491
H  -0.18329  4.32468  3.18874
C   2.35547  3.46630  3.93350
H   2.31138  4.56961  3.96005
H   2.01469  3.08952  4.91393
H   3.41184  3.17650  3.80668
C   4.21791  2.31981 -1.53178
H   4.18794  1.26628 -1.20393
C   5.66423  2.82933 -1.32276
H   5.96932  2.75915 -0.26491
H   6.37601  2.23705 -1.92440
H   5.75830  3.88618 -1.62924
C   3.83965  2.33488 -3.02707
H   3.86226  3.35477 -3.44972
H   4.56027  1.72774 -3.60228
H   2.83611  1.91015 -3.18157
```

$Ar^{iPr_4}PO_2$

```
SCF (BP86) Energy = -1322.53153611
Enthalpy 0K = -1321.951074
Enthalpy 298K = -1321.914073
Free Energy 298K = -1322.021960
Lowest Frequency = 7.5847 cm-1
Second Frequency = 14.3601 cm-1
SCF (Bnz) Energy = -1322.53546362
SCF (BP86-D3BJ) Energy = -
1322.70480916
SCF (BS2) Energy = -1657.68892882
```

```
P   0.00005 -0.00004 -1.50113
O   0.00016  1.36960 -2.14588
C  -0.00002  0.00001  0.32952
C  -1.24436  0.00005  1.00222
C  -1.21991  0.00007  2.41197
H  -2.16839  0.00012  2.95841
C  -0.00000  0.00004  3.10656
H  -0.00000  0.00005  4.20157
C   1.21990 -0.00001  2.41197
H   2.16838 -0.00004  2.95840
C   1.24433 -0.00002  1.00222
C  -2.53242  0.00008  0.22485
C  -3.13959  1.24058 -0.13047
C  -4.35539  1.21218 -0.84017
H  -4.83474  2.15608 -1.12010
C  -4.96035  0.00015 -1.19260
H  -5.90572  0.00018 -1.74586
C  -4.35552 -1.21190 -0.84006
H  -4.83497 -2.15578 -1.11993
C  -3.13973 -1.24037 -0.13036
C   2.53240 -0.00007  0.22485
C   3.13956 -1.24057 -0.13047
C   4.35538 -1.21219 -0.84012
H   4.83472 -2.15610 -1.12006
C   4.96037 -0.00017 -1.19252
H   5.90577 -0.00021 -1.74574
C   4.35555  1.21189 -0.84000
H   4.83502  2.15577 -1.11985
C   3.13973  1.24038 -0.13034
C  -2.53570  2.58748  0.27503
H  -1.51316  2.39954  0.64704
C  -2.41469  3.56626 -0.91267
H  -1.81252  3.12630 -1.72297
H  -1.92391  4.49888 -0.58411
H  -3.40288  3.84330 -1.31995
C  -3.34322  3.21459  1.43581
H  -4.38350  3.42045  1.12762
H  -2.88923  4.16960  1.75344
H  -3.38068  2.54470  2.31173
C  -2.53598 -2.58731  0.27524
H  -1.51343 -2.39945  0.64726
C  -2.41503 -3.56618 -0.91239
H  -3.40324 -3.84315 -1.31968
H  -1.92436 -4.49882 -0.58376
H  -1.81281 -3.12633 -1.72271
C  -3.34357 -3.21427  1.43605
H  -3.38099 -2.54432  2.31192
H  -2.88969 -4.16930  1.75375
H  -4.38387 -3.42005  1.12785
C   2.53562 -2.58747  0.27501
H   1.51301 -2.39952  0.64681
C   2.41484 -3.56634 -0.91263
H   1.81283 -3.12643 -1.72308
H   1.92398 -4.49892 -0.58410
H   3.40310 -3.84343 -1.31970
C   3.34294 -3.21447  1.43599
H   4.38328 -3.42032  1.12800
H   2.88892 -4.16948  1.75359
H   3.38021 -2.54453  2.31187
C   2.53597  2.58733  0.27522
H   1.51341  2.39947  0.64720
C   3.34353  3.21430  1.43605
H   3.38091  2.54438  2.31194
H   2.88963  4.16934  1.75371
H   4.38384  3.42008  1.12788
C   2.41508  3.56617 -0.91243
H   3.40331  3.84314 -1.31968
H   1.92438  4.49882 -0.58385
H   1.81289  3.12630 -1.72277
O   0.00001 -1.36973 -2.14577
```

Section 8: NMR Spectra of Compounds 1-3

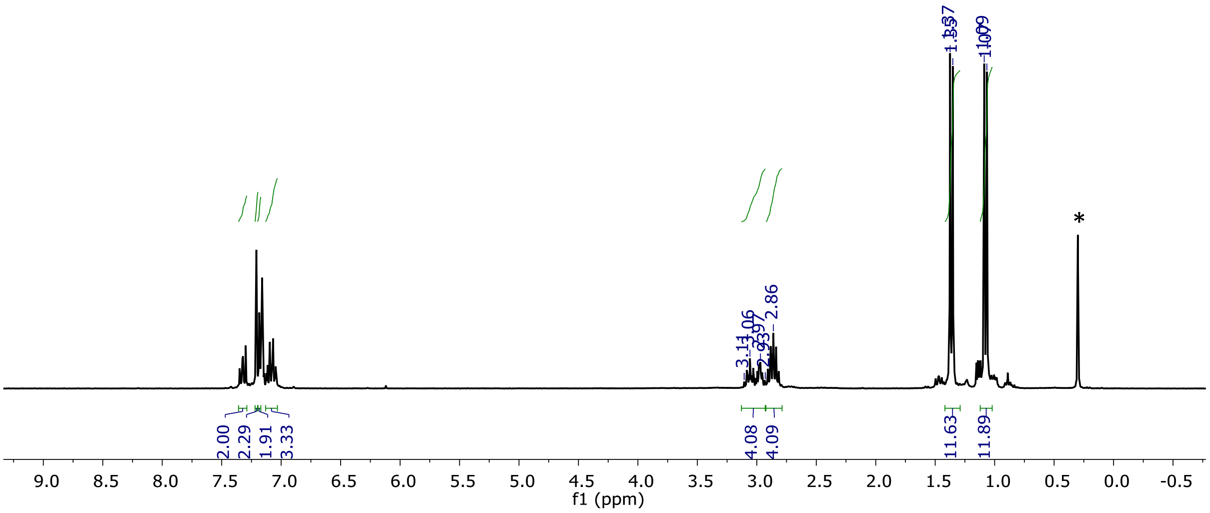

Figure S20: ^{1}H NMR spectrum of compound **1**, silicon grease marked with *

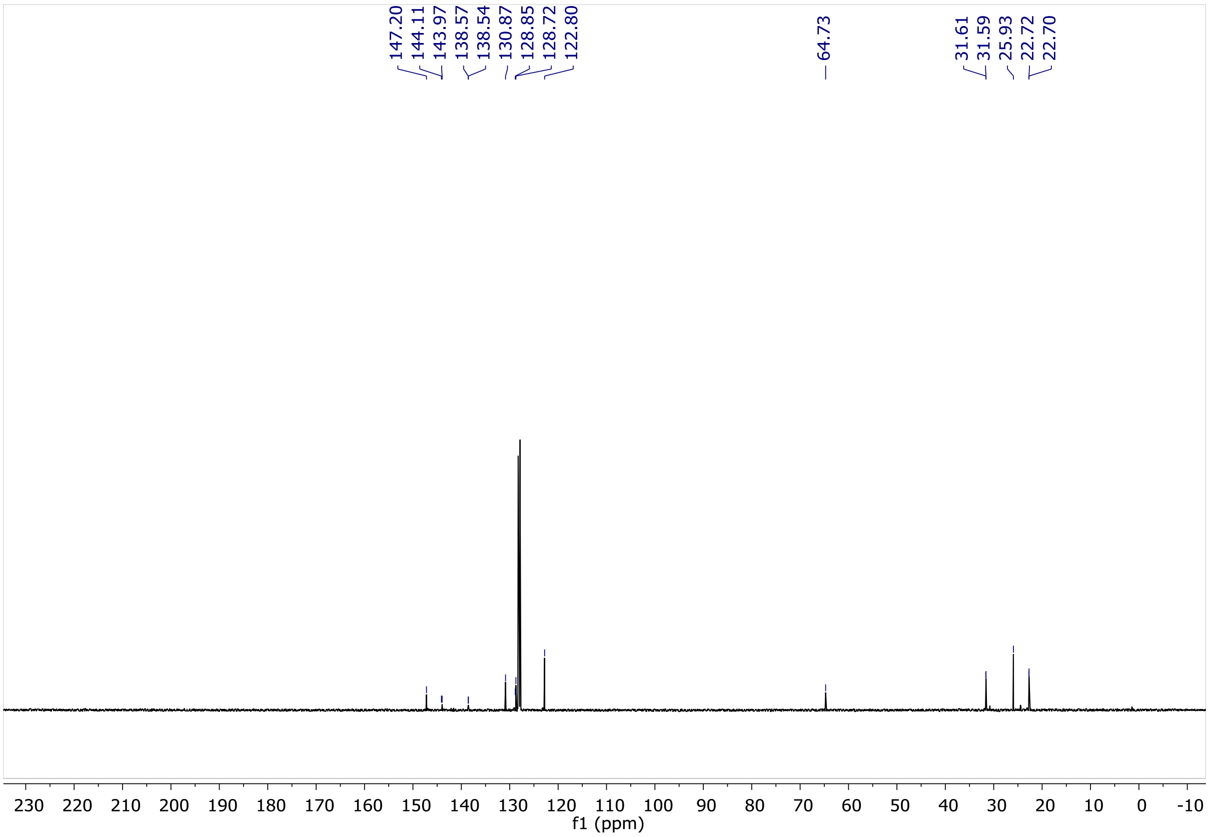

Figure S21: ^{13}C NMR spectrum of compound **1**

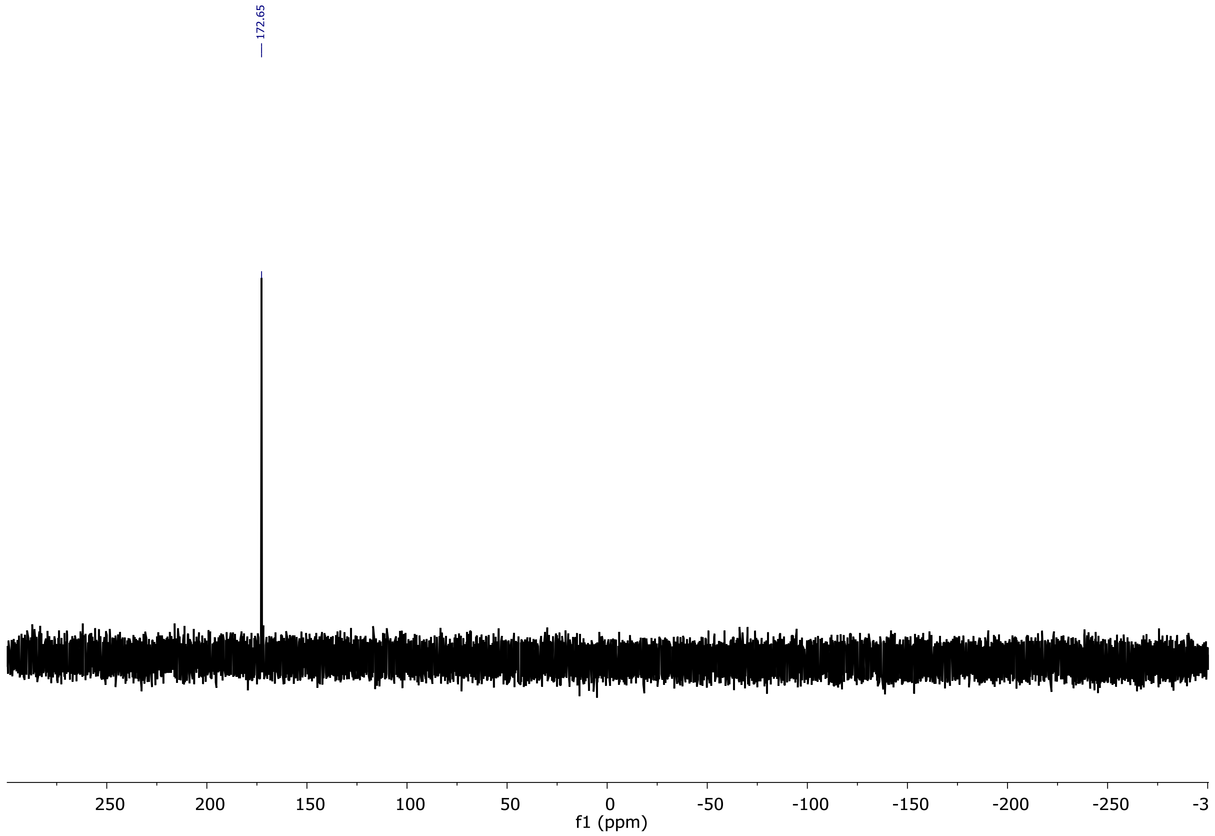

Figure S22: ^{31}P NMR spectrum of compound **1**

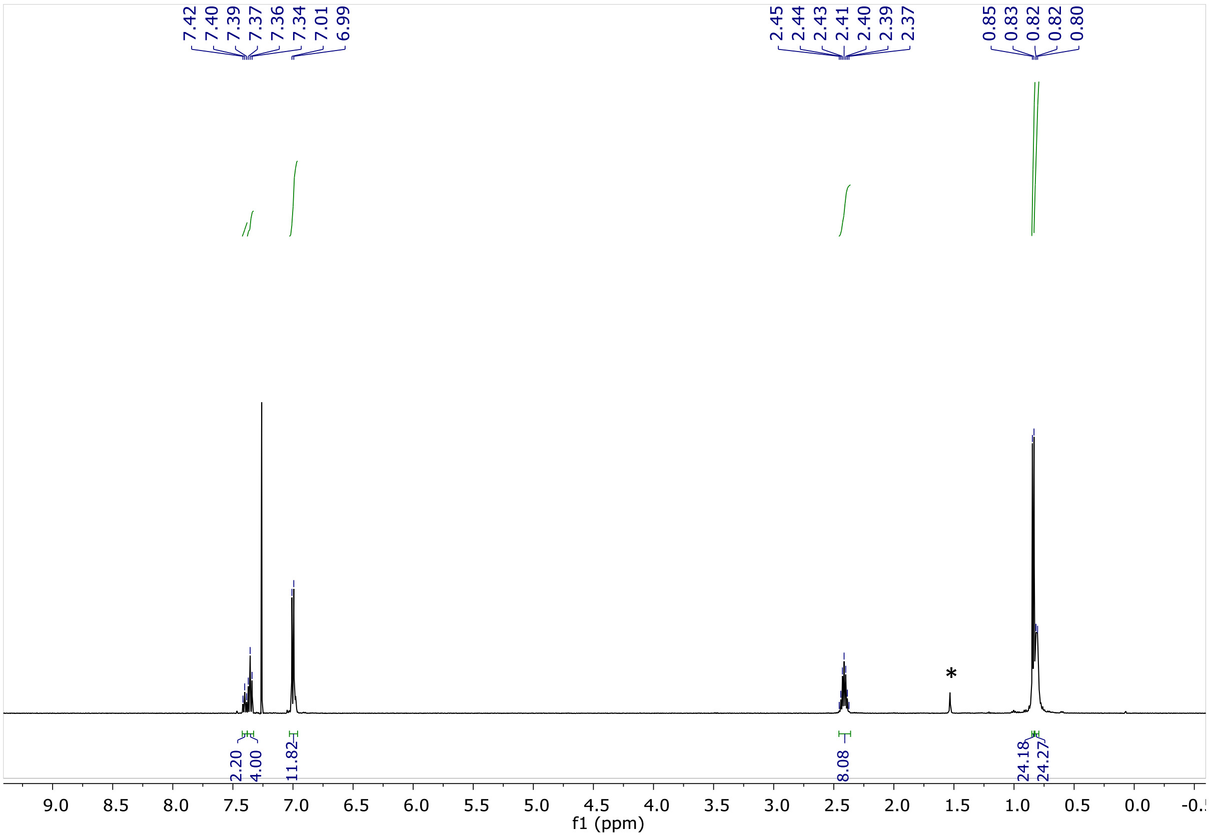

Figure S23: ^{1}H NMR spectrum of compound **2**, H_2O marked with *

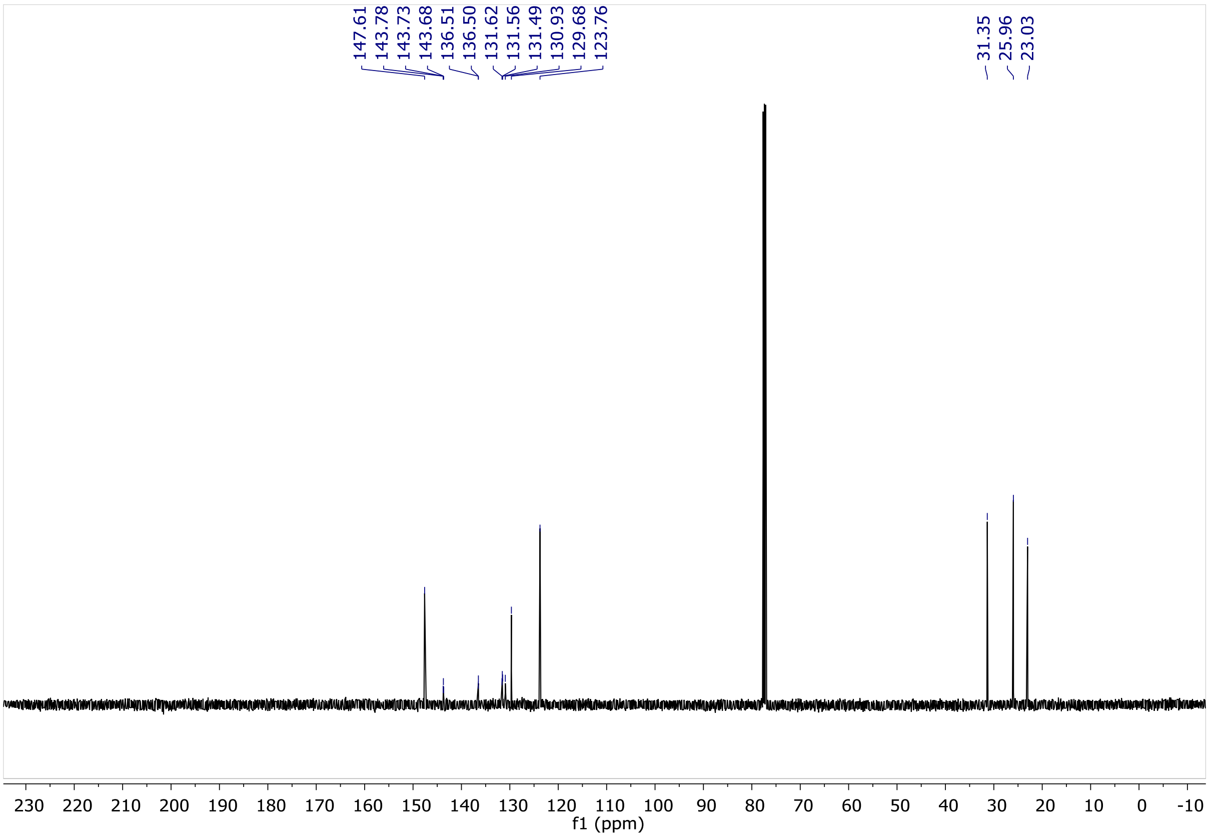

Figure S24: ^{13}C NMR spectrum of compound **2**

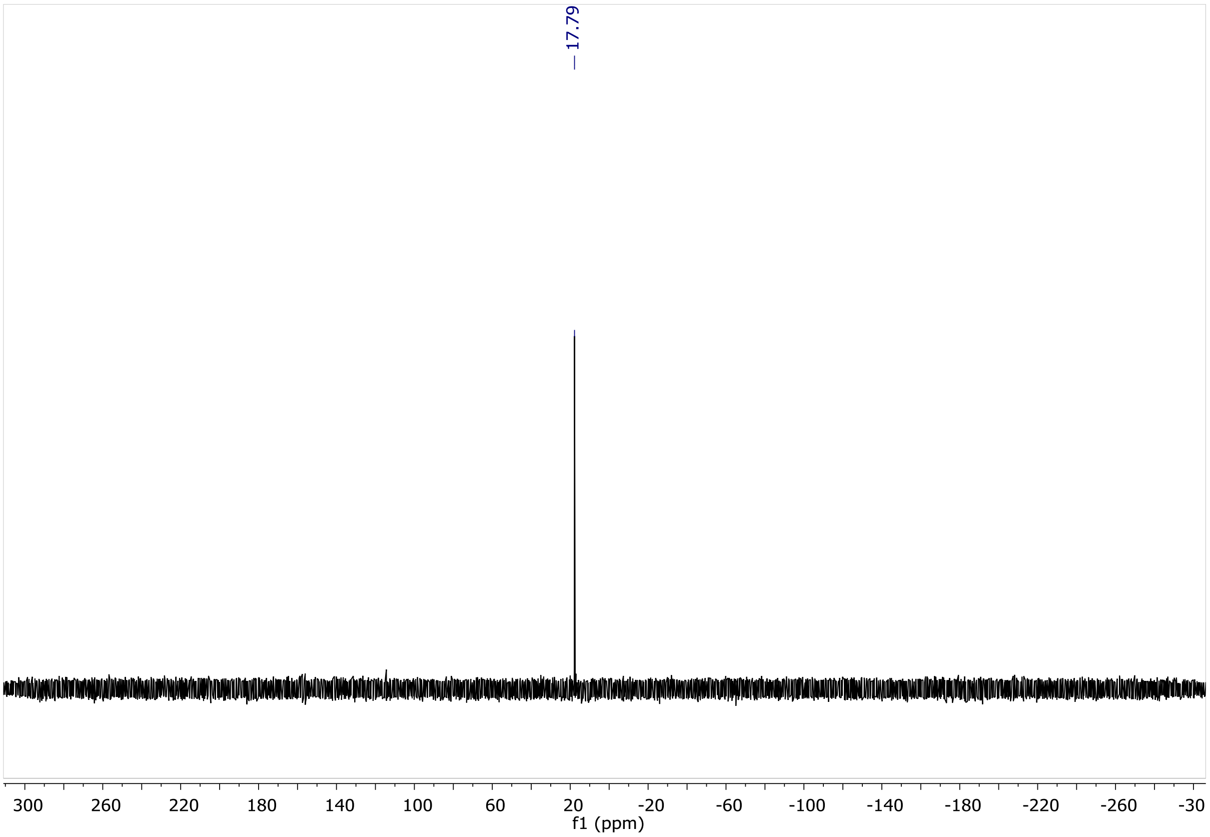

Figure S25: ^{31}P NMR spectrum of compound **2**

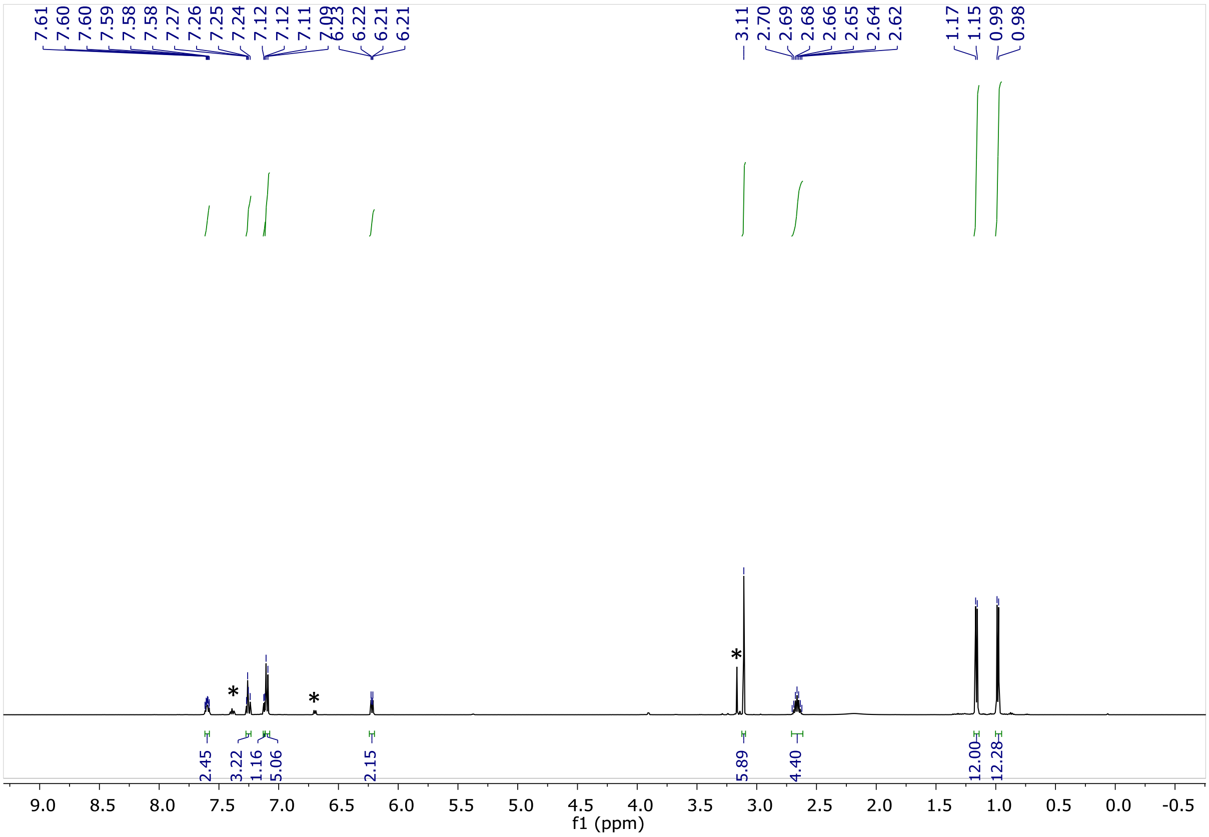

Figure S26: ^{1}H NMR spectrum of compound **3**, excess DMAP indicated with *

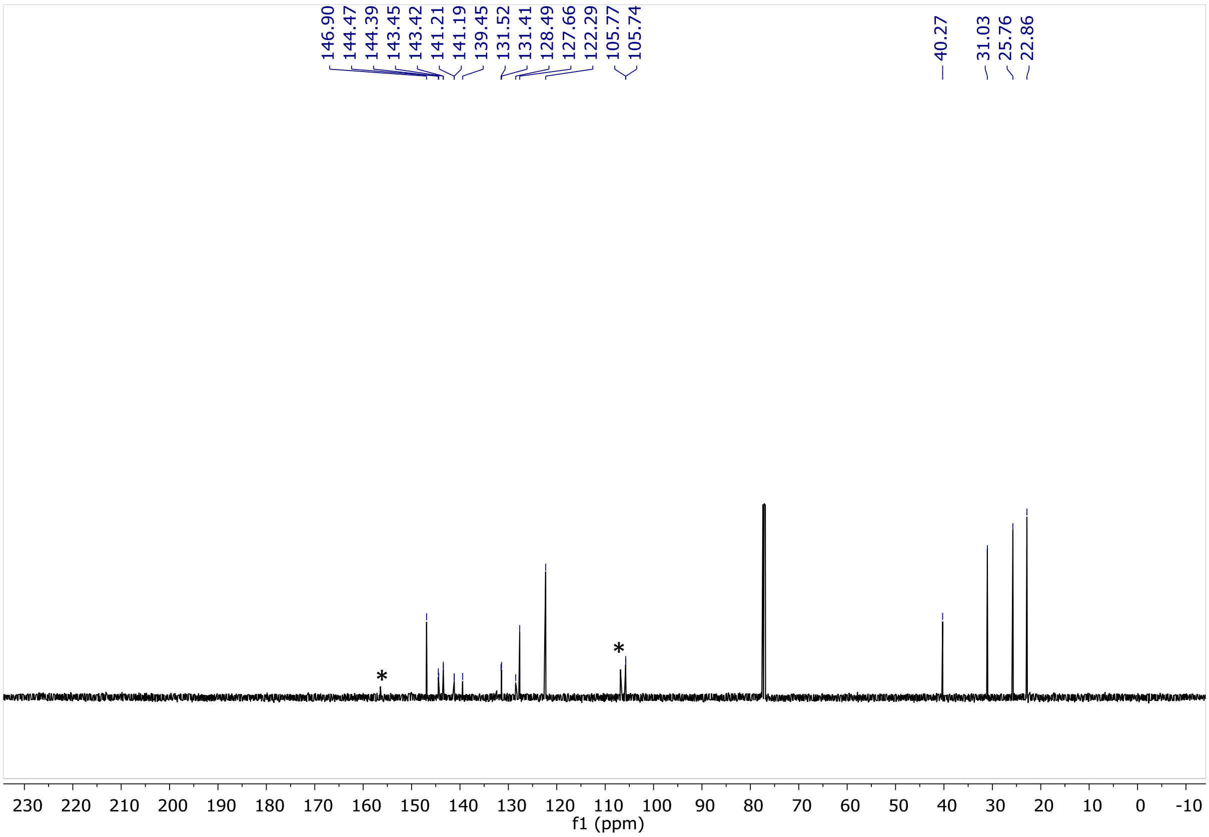

Figure S27: ^{13}C NMR spectrum of compound **3** excess DMAP indicated with *

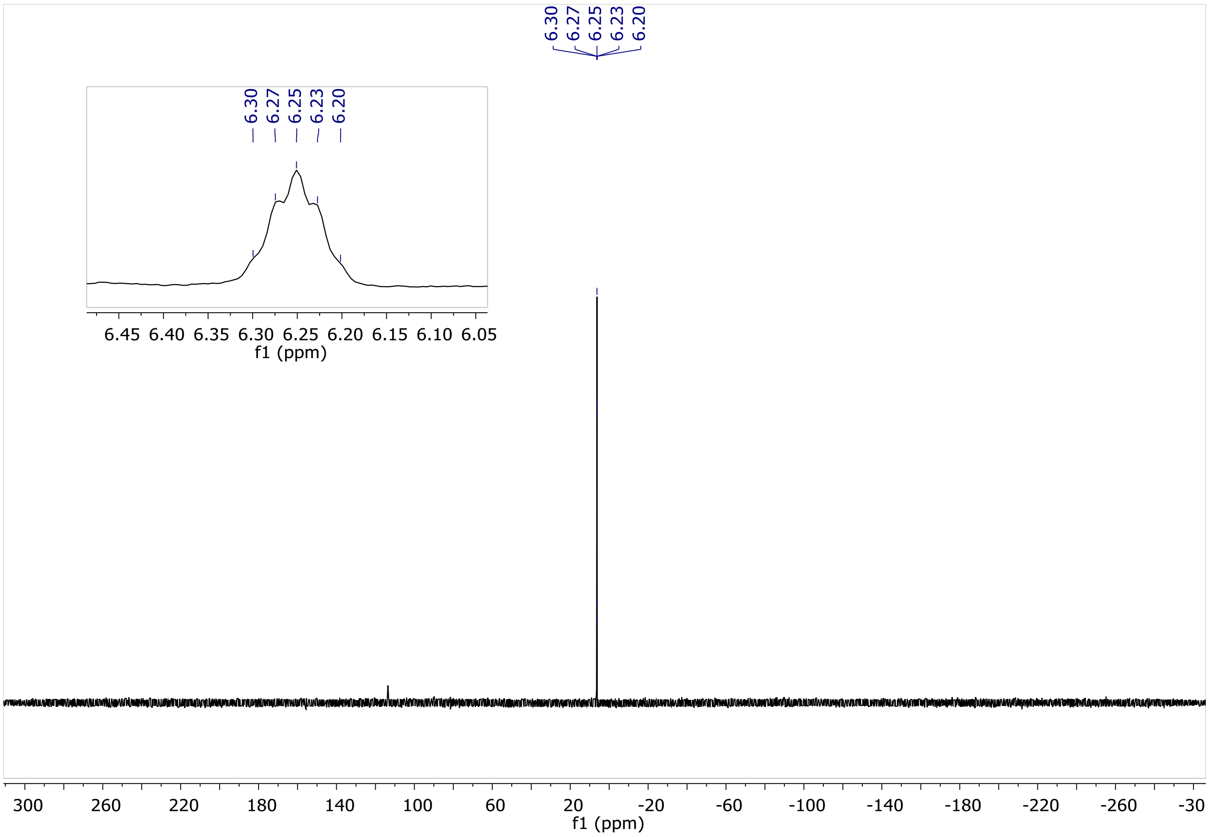

Figure S28: ^{31}P NMR spectrum of compound **3** with an inset (6.5-6.0 ppm) to show the multiplicity of the observed resonance

Section 9: IR Data for Compounds 2 and 3

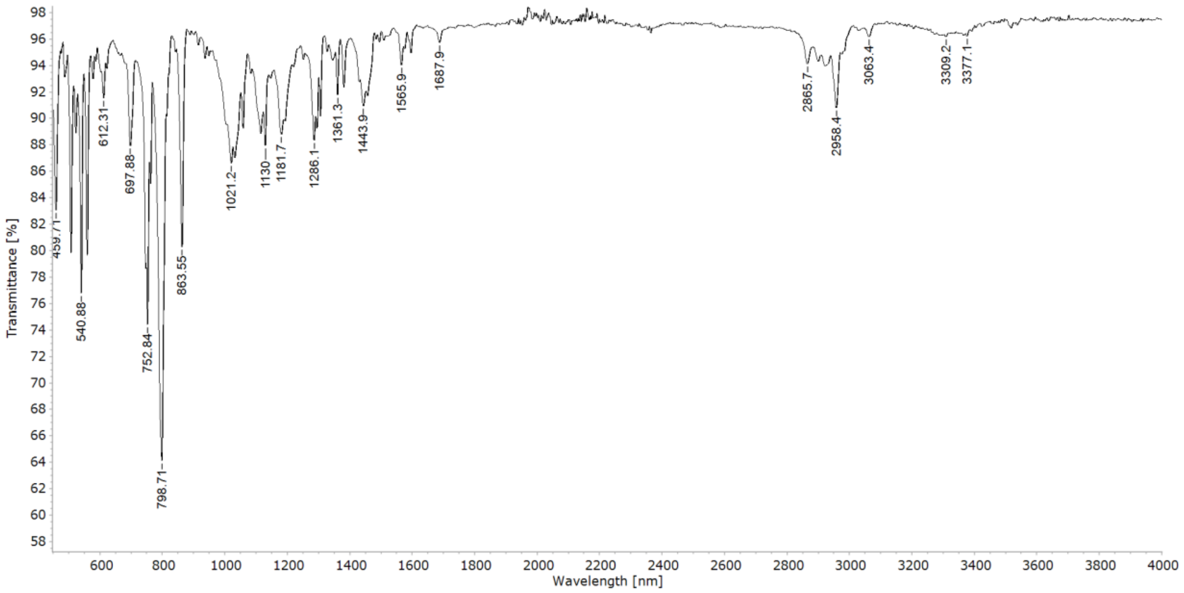

Figure S29: IR spectrum of compound **2** with selected peaks labelled

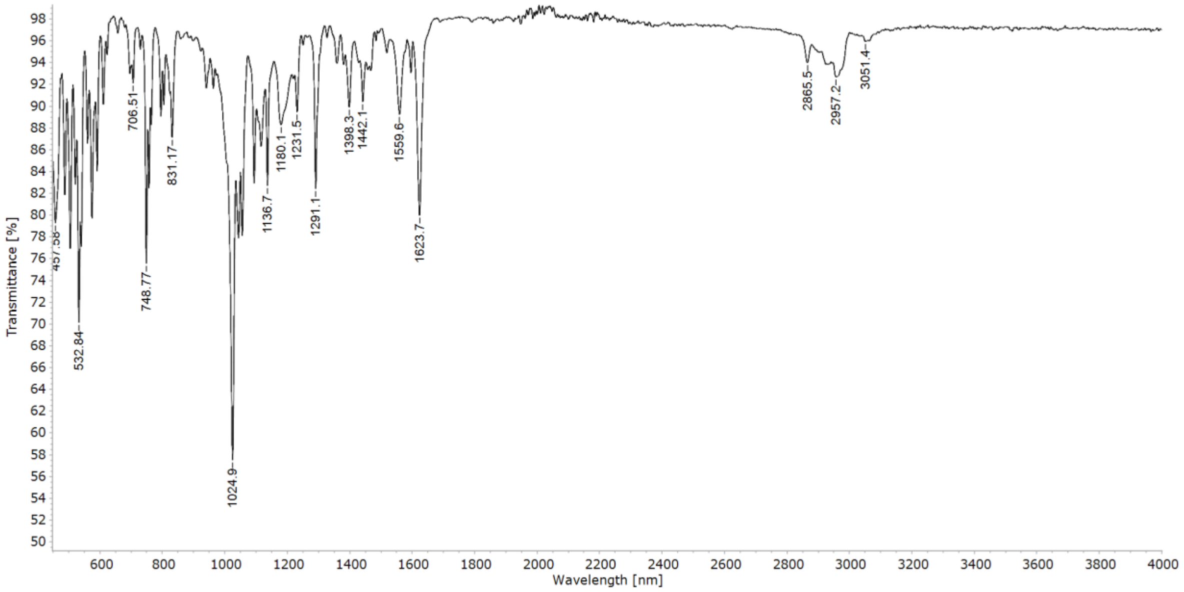

Figure S30: IR spectrum for compound **3** with selected peaks labelled

Section 10: References

1. B. R. Barnett, C. C. Mokhtarzadeh, J. S. Figueroa, P. Lummis, S. Wang, J. D. Queen, J. Gavenonis, N. Schüwer, T. D. Tilley, J. N. Boynton, P. P. Power, T. B. Ditri, N. Weidemann, B. R. Barnett, D. W. Agnew, J. S. Figueroa, P. W. Smith, T. B. Ditri, B. R. Barnett, A. E. Carpenter, C. C. Mokhtarzadeh, D. W. Agnew, J. S. Figueroa, P. W. Smith, J. K. Pratt, P. P. Power, N. D. Mendelson, J. S. Figueroa, J. D. Queen, P. P. Power, D. W. Agnew, A. E. Carpenter, J. S. Figueroa, in *Inorg. Synth.*, **2018**, pp. 85-122.
2. Dolomanov, O.V., Bourhis, L.J., Gildea, R.J, Howard, J.A.K. & Puschmann, H., *J. Appl. Cryst.*, **2009**, *42*, 339-341.
3. Sheldrick, G.M. (2015). *Acta Cryst. A,* **2015**, *71*, 3-8.
4. Sheldrick, G.M. (2015). *Acta Cryst. C71*, **2015**, *71*, 3-8.
5. Gaussian 16, Revision C.01, Frisch, M. J.; Trucks, G. W.; Schlegel, H. B.; Scuseria, G. E.; Robb, M. A.; Cheeseman, J. R.; Scalmani, G.; Barone, V.; Petersson, G. A.; Nakatsuji, H.; Li, X.; Caricato, M.; Marenich, A. V.; Bloino, J.; Janesko, B. G.; Gomperts, R.; Mennucci, B.; Hratchian, H. P.; Ortiz, J. V.; Izmaylov, A. F.; Sonnenberg, J. L.; Williams-Young, D.; Ding, F.; Lipparini, F.; Egidi, F.; Goings, J.; Peng, B.; Petrone, A.; Henderson, T.; Ranasinghe, D.; Zakrzewski, V. G.; Gao, J.; Rega, N.; Zheng, G.; Liang, W.; Hada, M.; Ehara, M.; Toyota, K.; Fukuda, R.; Hasegawa, J.; Ishida, M.; Nakajima, T.; Honda, Y.; Kitao, O.; Nakai, H.; Vreven, T.; Throssell, K.; Montgomery, J. A., Jr.; Peralta, J. E.; Ogliaro, F.; Bearpark, M. J.; Heyd, J. J.; Brothers, E. N.; Kudin, K. N.; Staroverov, V. N.; Keith, T. A.; Kobayashi, R.; Normand, J.; Raghavachari, K.; Rendell, A. P.; Burant, J. C.; Iyengar, S. S.; Tomasi, J.; Cossi, M.; Millam, J. M.; Klene, M.; Adamo, C.; Cammi, R.; Ochterski, J. W.; Martin, R. L.; Morokuma, K.; Farkas, O.; Foresman, J. B.; Fox, D. J. Gaussian, Inc., Wallingford CT, 2016.
6. Andrae, D., Häußermann, U., Dolg, M., Stoll, H., Preuß, H., *Theor. Chim. Acta*, **1990**, *7*, 123−141.
7. (a) Hariharan, P. C., Pople, J. A. *Theor. Chim. Acta*, **1973**, *28*, 213−222. (b) Hehre, W. J., Ditchfield, R., Pople, J. A. *J. Chem. Phys.*, **1972**, *56*, 2257.
8. Becke, A. D. *Phys. Rev. A: At., Mol., Opt. Phys.*, **1988**, *38*, 3098. (b) Perdew, J. P. *Phys. Rev. B: Condens. Matter Mater. Phys.*, **1986**, *33*, 8822−8824.
9. Tomasi, J., Mennucci, B., Cammi, R. *Chem. Rev.*, **2005**, *105*, 2999−3094.
10. S. Grimme, S. Ehrlich and L. Goerigk, *J. Comp. Chem.*, **2011**, *32*, 1456-1465.
